# Functions of Huntingtin in Germ Layer Specification and Organogenesis

**DOI:** 10.1371/journal.pone.0072698

**Published:** 2013-08-13

**Authors:** Giang D. Nguyen, Aldrin E. Molero, Solen Gokhan, Mark F. Mehler

**Affiliations:** 1 Roslyn and Leslie Goldstein Laboratory for Stem Cell Biology and Regenerative Medicine, Albert Einstein College of Medicine, Bronx, New York, United States of America; 2 Institute for Brain Disorders and Neural Regeneration, Albert Einstein College of Medicine, Bronx, New York, United States of America; 3 Department of Neurology, Albert Einstein College of Medicine, Bronx, New York, United States of America; 4 Department of Neuroscience, Albert Einstein College of Medicine, Bronx, New York, United States of America; 5 Department of Psychiatry and Behavioral Sciences, Albert Einstein College of Medicine, Bronx, New York, United States of America; 6 Rose F. Kennedy Center for Research on Intellectual and Developmental Disabilities, Albert Einstein College of Medicine, Bronx, New York, United States of America; 7 Einstein Cancer Center, Albert Einstein College of Medicine, Bronx, New York, United States of America; 8 Ruth L. and David S. Gottesman Institute for Stem Cell Biology and Regenerative Medicine, Albert Einstein College of Medicine, Bronx, New York, United States of America; 9 Center for Epigenomics, Albert Einstein College of Medicine, Bronx, New York, United States of America; 10 Institute for Aging Research, Albert Einstein College of Medicine, Bronx, New York, United States of America; Instituto de Medicina Molecular, Portugal

## Abstract

Huntington’s disease (HD) is a neurodegenerative disease caused by abnormal polyglutamine expansion in the huntingtin protein (Htt). Although both Htt and the HD pathogenic mutation (mHtt) are implicated in early developmental events, their individual involvement has not been adequately explored. In order to better define the developmental functions and pathological consequences of the normal and mutant proteins, respectively, we employed embryonic stem cell (ESC) expansion, differentiation and induction experiments using huntingtin knock-out (KO) and mutant huntingtin knock-in (Q111) mouse ESC lines. In KO ESCs, we observed impairments in the spontaneous specification and survival of ectodermal and mesodermal lineages during embryoid body formation and under inductive conditions using retinoic acid and Wnt3A, respectively. Ablation of BAX improves cell survival, but failed to correct defects in germ layer specification. In addition, we observed ensuing impairments in the specification and maturation of neural, hepatic, pancreatic and cardiomyocyte lineages. These developmental deficits occurred in concert with alterations in Notch, Hes1 and STAT3 signaling pathways. Moreover, in Q111 ESCs, we observed differential developmental stage-specific alterations in lineage specification and maturation. We also observed changes in Notch/STAT3 expression and activation. Our observations underscore essential roles of Htt in the specification of ectoderm, endoderm and mesoderm, in the specification of neural and non-neural organ-specific lineages, as well as cell survival during early embryogenesis. Remarkably, these developmental events are differentially deregulated by mHtt, raising the possibility that HD-associated early developmental impairments may contribute not only to region-specific neurodegeneration, but also to non-neural co-morbidities.

## Introduction

Huntington’s disease (HD) is an autosomal dominant genetic disorder caused by abnormal CAG expansion in exon 1 of the huntingtin gene (m*htt*) and pathologically characterized by progressive degeneration of striatal and cortical neurons [1]. Clinical hallmarks of HD include the adult onset of characteristic neuropsychiatric and motor abnormalities. Since the first published characterization of HD by George Huntington in 1872, studies of HD pathogenesis have predominantly focused on interrogating disease stages exhibiting overt clinical signs and symptoms. Indeed, the functional pleiotropism of the huntingtin protein (Htt), which ranges from transcriptional regulation to anti-apoptotic functions, as well as the adverse effects of mHtt have been thoroughly studied in adult life [2–4].

However, several recent studies have suggested that Htt also plays essential roles during early embryogenesis. Targeted deletion of *htt* in mice (KO) resulted in excessive cell death in the epiblast and severe developmental defects such as head-fold involution, a shortened primitive streak and absence of the embryonic organizer, culminating in embryonic lethality as early as embryonic day 6.5 (E6.5) [5–8]. In addition, silencing of *htt* in progenitor cells of the ventricular zone from E14.5 has also been shown to alter their lineage commitment associated with enhanced cell death [9]. In addition, analysis of aggregation chimeras with htt^-/-^ ESCs revealed that Htt is essential for neural development in selective brain structures, particularly the striatum [10]. The findings of the *in vivo* ablation studies suggest that Htt may play critical roles in germ layer specification and region-specific neurogenesis. However, it remains unclear whether the pathogenic HD mutation may impair these early developmental events. Indeed, our group has recently demonstrated an array of developmental impairments in the specification and maturation of striatal medium spiny neurons (MSNs) in a m*htt* knock-in mouse model (Q111) as early as E13.5 [11]. Therefore, it is plausible that mHtt may also impair not only germ layer specification, but also organogenesis, and thus contribute to HD-associated systemic co-morbidities.

In this study, we examined the roles of Htt and the potential adverse effects of mHtt during early embryonic development. We analyzed huntingtin knock-out (KO) and Q111 ESCs utilizing well established ESC culture paradigms to recapitulate early developmental events [12]. We hypothesized that Htt plays important spatial and temporal roles during embryogenesis and that mHtt differentially alters these key developmental events.

## Results

Htt is not required for the maintenance of undifferentiated ESCs, but is important for specification and survival of ectoderm, endoderm and mesoderm, whereas mHtt impairs spontaneous ESC differentiation and differentially alters derivatives of these germ layers

**Figure 1:**
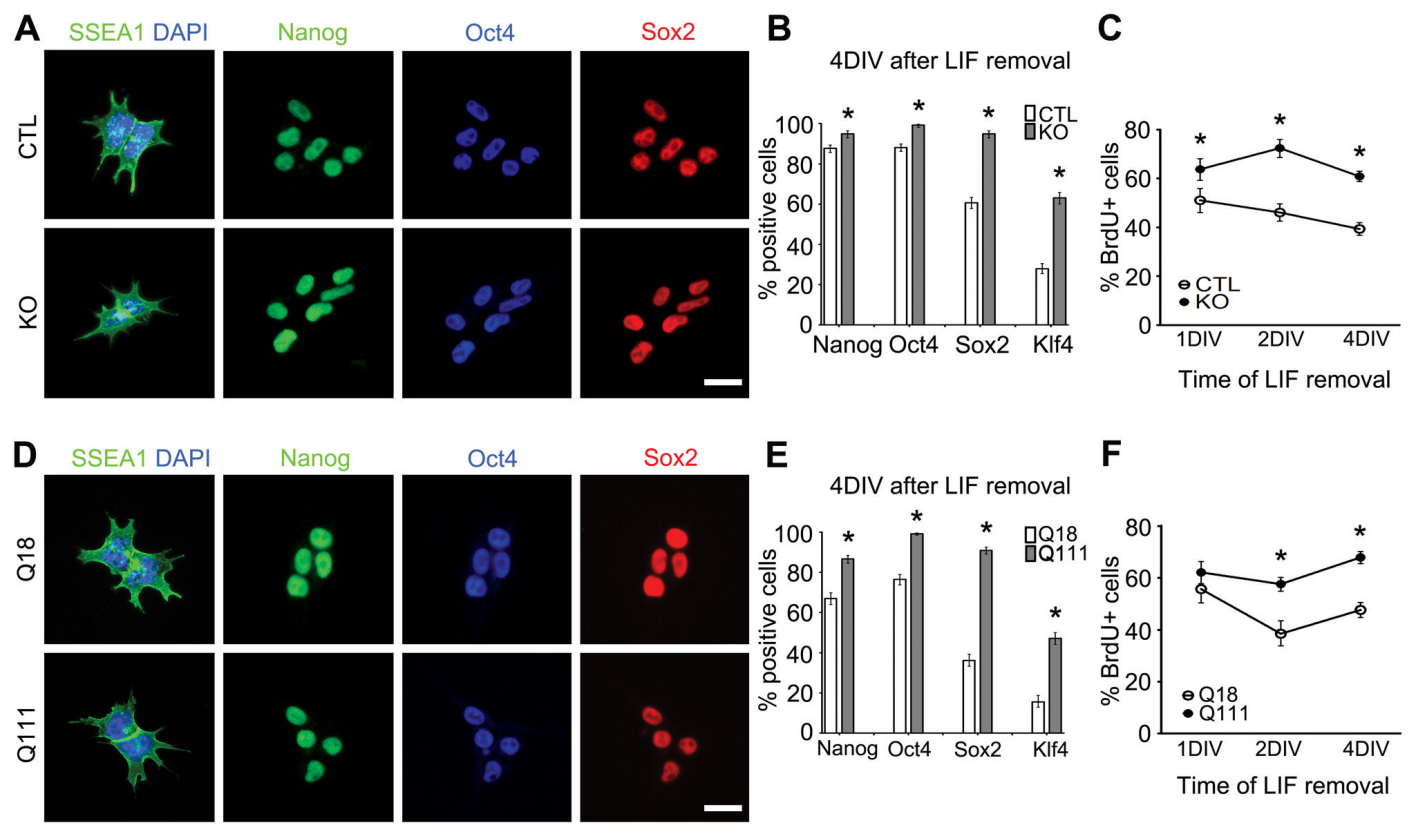
mHtt impairs the spontaneous differentiation of ESCs in ways analogous to Htt ablation. (A, D) Immunofluorescence analysis of the ESC marker (SSEA1) and the pluripotency factors (Nanog, Oct4, Sox2) in undifferentiated ESC maintained with LIF. (B, E) Quantification of Nanog+, Oct4+, Sox2+ (n=1203, 995, 1138 and 608 for CTL, KO, Q18 and Q111, respectively) and Klf4+ (n=1228, 592, 1113 and 1112 for CTL, KO, Q18 and Q111, respectively) cells present in ESCs after 4 DIV following removal of LIF. (C, F) ESC cultures pulsed with BrdU for 4 hrs in media without LIF. The ESCs were then fixed at 1DIV, 2DIV, and 4DIV and quantification of BrdU+ cells was assessed at these three time points (n=1426, 1162, 1571 and 2033 for CTL, KO, Q18 and Q111, respectively). All error bars represent ±95% CI; *p-values < 0.0001 unless otherwise noted. Scale bar = 20 µm.

Our group recently reported developmental alterations in the expression profiles of Nanog and Sox2 in the striatal generative zone and mantle region of the Q111 mouse brain [11]. These factors, together with Oct4 and Klf4, form the core pluripotency network that is critical for the maintenance and differentiation of ESCs [13]. To determine whether Htt is required for the regulation of pluripotency factors and consequentially for the maintenance of undifferentiated ESCs, we compared Hdh^ex4,5/ex4,5^ ESCs [7,14,15], hereby referred to as KO ESCs, with wild-type ESCs (CTL ESCs). To further investigate the effects of the pathogenic HD mutation on these functions, we compared m*htt* knock-in ESCs, hereby referred to as Q111 ESCs, which carries an expanded polyglutamine tract (111 glutamines), with wild type *htt* knock-in ESCs, hereby referred to as Q18, which conversely carries a normal polyglutamine tract (18 glutamines) [15,16]. There were no differences in the expression profiles of the pluripotency factors, Nanog, Oct4, Sox2 and Klf4, and the ESC marker, SSEA1, as well as KI67 and phosphorylated histone H3 (pHisH3), markers for dividing cells and the G2/M-phase of the cell cycle, respectively, in KO ESCs versus CTL ESCs and in Q111 ESCs versus Q18 ESCs (Figure 1A, D; Figure S1A–F). These observations indicate that Htt is not required for maintenance of undifferentiated ESCs and the regulation of the core pluripotency factors, and mHtt does not alter these earliest developmental functions.

**Figure 2:**
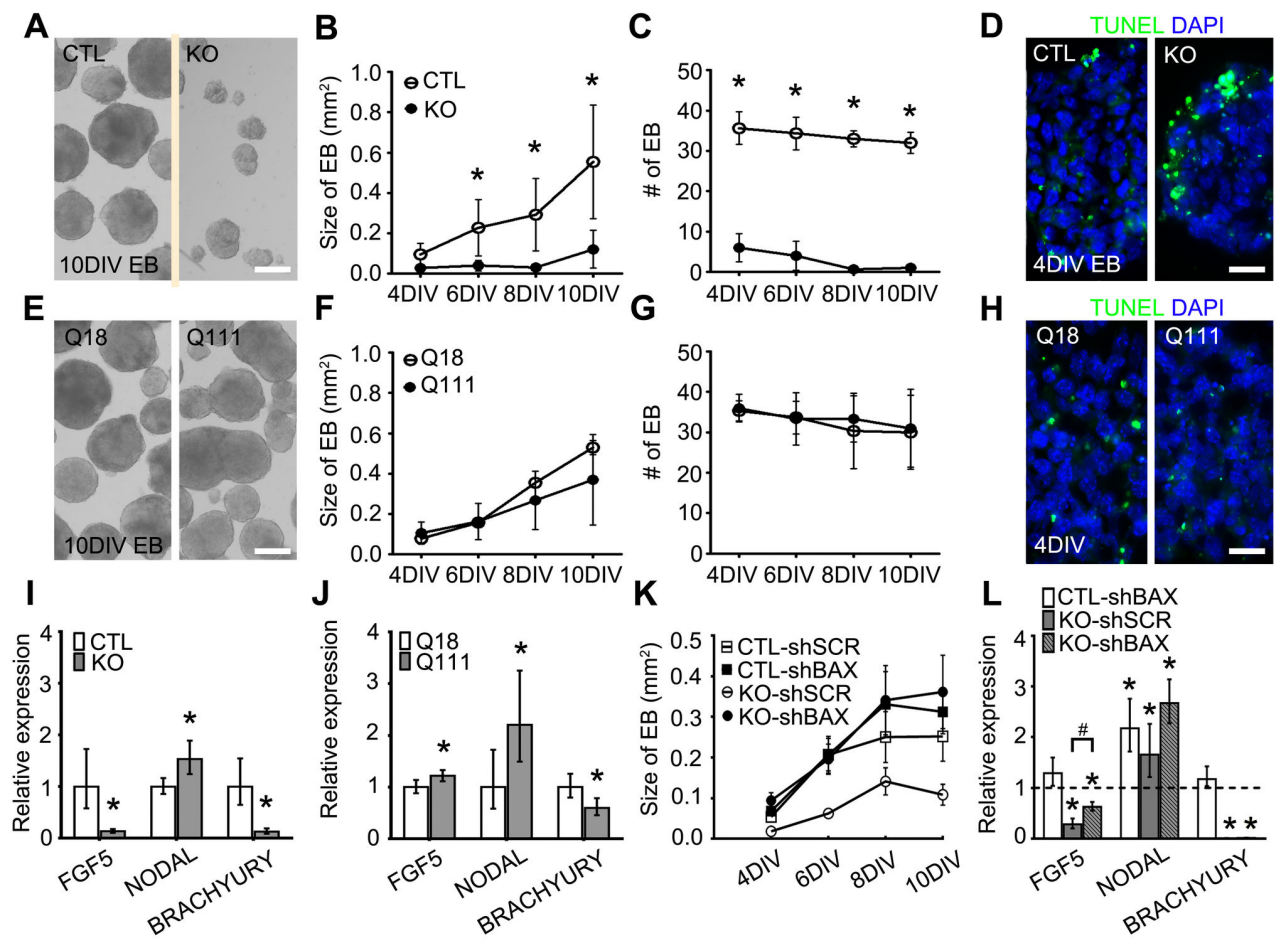
Specification and survival of three germ layers requires Htt whereas mHtt differentially impairs these processes. (A) Representative images of different sizes of CTL and KO EBs at 10 DIV. (B, C) Quantification of the size and number of CTL and KO EBs at 4DIV, 6DIV, 8DIV and 10DIV. Error bars represent ±SEM; *p<0.0001. (D) Representative images of TUNEL assay in CTL and KO EB at 4 DIV. (E) Representative images of different sizes of Q18 and Q111 EBs at 10 DIV. (F, G) Quantification of the size and number of Q18 and Q111 EBs at 4DIV, 6DIV, 8DIV and 10DIV. Error bars represent ±SEM; *p<0.0001. (H) Representative images of TUNEL assay in Q18 and Q111 EBs at 4 DIV. (I, J) QPCR analysis of developmental markers representing the ectodermal (FGF5), endodermal (NODAL) and mesodermal (BRACHYURY) germ layers. Error bars represent ±95% CI; *p<0.0001. (K) Quantification of the size of CTL and KO EBs formed after knocking down BAX using lentiviral transfection with a double short hairpin RNA (shRNA). (L) QPCR analysis of germ layer developmental markers in CTL-shSCR, CTL-shBAX, KO-shSCR and KO-shBAX specimens. Error bars represent ±95% CI; *p<0.0001, #p<0.0001 unless otherwise noted. Scale bar = 20 µm (A, E); 200 µm (D, H).

We next investigated whether Htt and mHtt are involved in ESC differentiation, by analyzing spontaneously differentiating ESCs obtained after removal of leukemia inhibitory factor (LIF), a critical factor for ESC maintenance [17]. Four days *in vitro* (DIV) following LIF removal, both KO ESCs and Q111 ESCs, respectively, exhibited constitutive cellular expression of the pluripotency factors in concert with persistent expression of BrdU, KI67 and pHisH3, as compared to the progressive downregulation of these developmental factors and cell cycle parameters in CTL ESCs and Q18 ESCs, respectively (all comparisons are statistically significant with p-values < 0.0001; Figure 1B, C, E and F; Figure S2A–J). These observations indicate that both the absence of Htt and the presence of mHtt may alter the capacity of ESCs to differentiate spontaneously by suppressing the downregulation of pluripotency factors and the active modulation of cell cycle progression. To further examine the possibility that Htt regulates the subsequent specification of the three cardinal germ layers, we assessed the specification of the derivatives of the three germ layers in ESC-derived embryoid bodies (EBs), which have been shown to partially recapitulate the process of gastrulation *in vivo* [18]. Compared to CTL, we observed a significant proportion of TUNEL+ dying cells in KO EBs as early as 4DIV (29.6% vs 9.2%, p-values < 0.0001), with severe reductions in both the number and size of KO EBs (DIV4: 6.0 vs 35.7, p-value = 0.00064; 0.028 mm^2^ vs 0.096 mm^2^, p-value < 0.0001; DIV6, 4 vs 34.3, p-value = 0.00063; 0.039 mm^2^ vs 0.227 mm^2^, p-value < 0.0001; DIV8, 1.0 vs 33.0, p-value < 0.0001; 0.032 mm^2^ vs 0.292 mm^2^, p-value < 0.0001; DIV10, 1.0 vs 32.0, p-value < 0.0001; 0.121 mm^2^ vs 0.554 mm^2^, p-value < 0.0001; respectively, Figure 2A–D). In contrast, there were no differences in the size and number of Q111 EBs as well as in the proportion of TUNEL+ cells as compared to Q18 EBs (Figure 2E–H). Further, gene expression analysis of *FGF5* (ectoderm), *Nodal* (endoderm) and *Brachyury* (mesoderm) revealed that both KO- and Q111-EBs, as compared to their respective controls, have significantly higher expression of *Nodal* (Fold change [Fc]: 1.533, p-value < 0.001; Fc: 2.204, p-value < 0.001; respectively) and lower expression of *Brachyury* (Fc: 0.131, p-value < 0.001; Fc: 0.596, p-value = 0.001; respectively). However, *FGF5* expression was significantly lower in KO EBs while it was increased in Q111 EBs (Fc: 0.138, p-value < 0.001; Fc: 1.216, p-value < 0.001; respectively; Figure 2I and J). These findings suggest that Htt plays important roles in maintaining the integrity of germ layer specification, and mHtt disrupts these developmental events.

Previous studies have shown that Htt has anti-apoptotic functions [19,20]. Given the fact that cell death was significantly increased in KO EBs, it is possible that impairments in germ layer specification may stem from differential profiles of cell survival of ESC-derived progenitor species. To investigate this possibility, we knocked down BAX, an upstream regulator of the caspase 3/9 apoptosis signaling cascade to attempt to enhance cell survival in KO EBs. Using this approach, there was a relative rescue of the proportion of TUNEL+ dying cells (KO-shSCR: 46.6%; KO-shBAX: 30.3%; CTL-shSCR: 26.0%; CTL-shBAX: 26.5%) and of the size of KO-shBAX EBs as compared to control EBs expressing a scrambled shRNA (CTL-shSCR) or BAX shRNA (CTL-shBAX) (Figure 2K and Figure S3). However, *Brachyury* gene expression in KO-shBAX EBs remained unchanged from KO-shSCR EBs and significantly reduced as compared to CTL-shSCR EBs (Fc: 0.012, p-value < 0.001). In addition, there was further enhancement of *Nodal* expression as compared to both CTL-shSCR and KO-shSCR EBs (Fc: 2.670, p-value < 0.001; Fc: 1.61, p-value < 0.001; respectively). Although *FGF5* expression showed a significant upregulation in KO-shBAX EBs as compared to KO-shSCR EBs (Fc: 2.12, p-value < 0.001), it remained significantly lower as compared to CTL-shSCR EBs (Fc: 0.62, p-value = 0.006; Figure 2L). The rescue of cell viability together with partial rescue of germ layer-associated gene expression suggests that Htt plays primary roles in cell survival during both ESC maintenance and germ layer specification.

### Htt is required for specification of mesendodermal progenitors and survival of neuroectodermal progenitors, whereas mHtt promotes precocious specification of neuroectodermal fate

**Figure 3:**
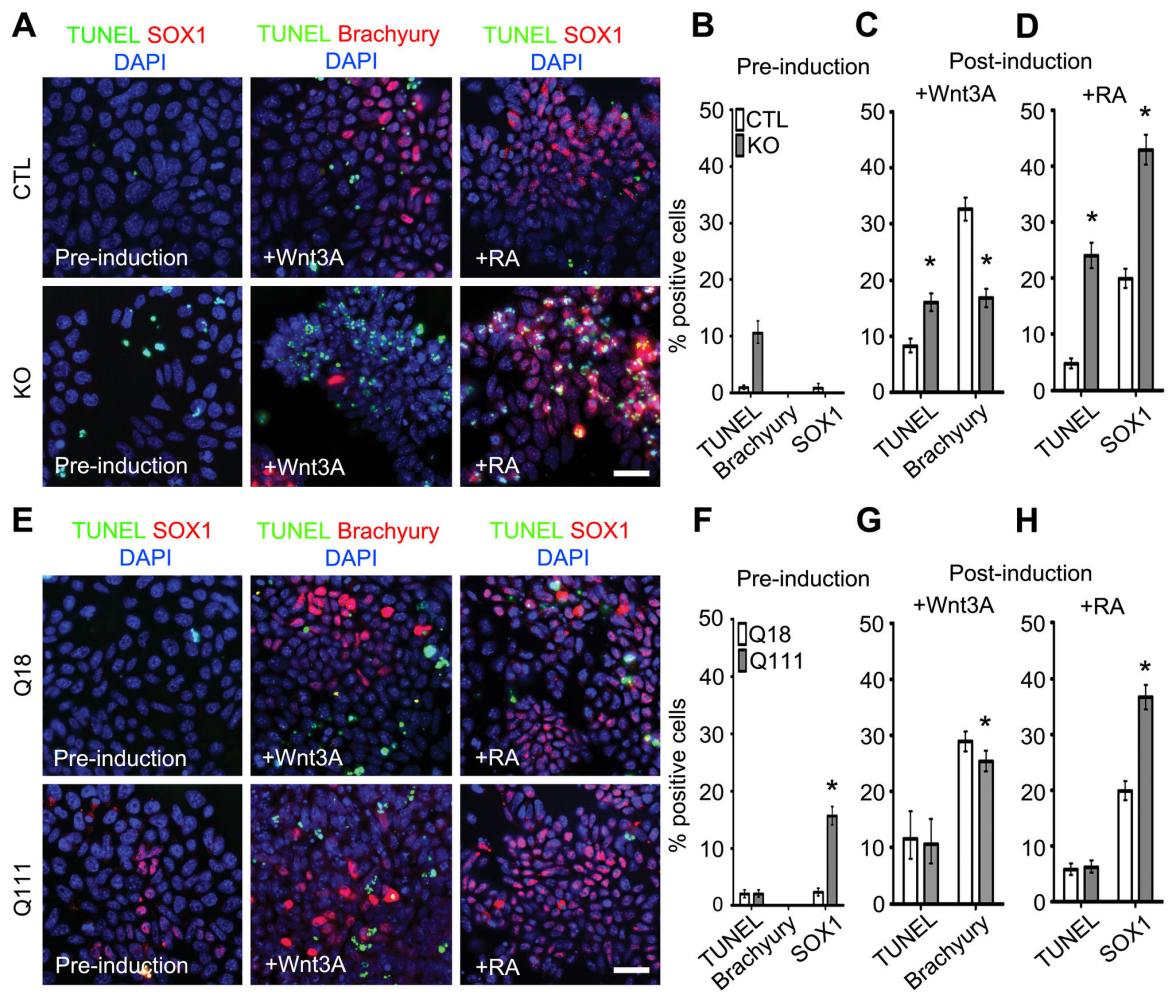
Induction and survival of mesendodermal and neuroectodermal progenitors requires Htt, whereas mHtt promotes neuroectodermal specification. (A) Representative images of immunofluorescence analyses of TUNEL, and Brachyury and SOX1 expression in CTL and KO ESCs pre- and post-induction. No Brachyury+ cells were detected pre-induction (data not shown). (B) Quantification of TUNEL+, Brachyury+ and SOX1+ cells pre-induction in CTL and KO ESCs (n=1509 and 928 for CTL and KO, respectively). (C) Quantification of TUNEL+ and Brachyury+ cells post- induction with Wnt3A in CTL and KO ESCs. (n=2065 and 2109 for CTL and KO, respectively.) (D) Quantification of TUNEL+ and SOX1+ cells post-induction with RA in CTL and KO ESCs (n=2149 and 1346 for CTL and KO, respectively). (E) Representative images of immunofluorescence analyses of TUNEL, and Brachyury and Sox1 expression in Q18 and Q111 ESCs pre- and post-induction. No Brachyury+ cells were detected at 48hr pre-induction (data not shown). (F) Quantification of TUNEL+, Brachyury+ and SOX1+ cells pre-induction in Q18 and Q111 ESCs (n=1608 and 1438 for Q18 and Q111, respectively). (G) Quantification of TUNEL+ and Brachyury+ cells post-induction with Wnt3A in Q18 and Q111 ESCs (n=2348 and 2169 for Q18 and Q111, respectively). (H) Quantification of TUNEL+ and SOX1+ cells post-induction with RA in Q18 and Q111 ESCs (n=1972 and 1859 for Q18 and Q111, respectively). All error bars represent ± 95% CI; *p<0.0001 unless otherwise noted. Scale bar = 20 µm.

During gastrulation, the posterior region of primitive ectoderm generates mesendodermal progenitors that subsequently give rise to definitive endoderm and mesoderm, whereas the anterior region generates neuroectodermal progenitors that give rise to the developing nervous system [21]. *In vitro* inductive paradigms using Wnt3A and retinoic acid (RA) in the absence of EB formation have been utilized to generate early mesendodermal and neuroectodermal cell types, respectively [22]. We employed this instructive experimental protocol to examine whether Htt plays a role in the early program of neuroectodermal and mesendodermal specification. Prior to Wnt3A induction, KO ESCs exhibited a significantly higher percentage of TUNEL+ dying cells as compared to CTL ESCs (0.9% vs 10.6%, p-value < 0.0001; Figure 3A and B). Following Wnt3A induction, KO ESCs contained a significantly lower percentage of Brachyury+ mesendodermal progenitors and a higher percentage of TUNEL+ cells as compared to CTL ESCs (TUNEL: 15.9% vs 8.2%, p-value < 0.0001; Brachyury: 16.7% vs 32.6%, p-value < 0.0001; Figure 3A and C), indicating that Htt regulates the specification, maintenance and survival of mesendodermal progenitors. Conversely, after RA induction, there was a higher percentage in SOX1+ neuroectodermal progenitors in KO ESCs as compared to CTL ESCs, in the background of significant cell death (SOX1: 42.9% vs 19.9%, p-value < 0.0001; TUNEL: 24.0% vs 4.7%, p-value < 0.0001; Figure 3A and D). The high percentage of TUNEL+ cell death may correspond to selective apoptosis of non-neural lineages.

By contrast, there were no changes in the proportion of TUNEL+ cells in Q111 versus Q18 ESCs when examined before and after Wnt3A induction; however, there was already a higher percentage of SOX1+ neuroectodermal progenitors in Q111 ESCs as compared to Q18 ESCs before RA induction (15.6% vs 2.3%, p-value < 0.0001; Figure 3E-F), and this increase persisted after RA induction (36.7% vs 19.9%, p-value < 0.0001; Figure 3H). Interestingly, there was a modest decrease in the percentage of Brachyury+ mesendodermal progenitors as compared to Q18 (Brachyury: 25.4% vs 28.9%, p-value = 0.0023; respectively, Figure 3E–G). These findings suggest that mHtt promotes precocious ESC-mediated neuroectodermal specification and impairs mesendodermal specification.

Htt is required for the differentiation of glutamatergic and GABAergic neurons and the specification and maturation of oligodendrocytes, whereas mHtt impairs GABAergic neuronal specification and promotes precocious oligodendrocyte maturation

**Figure 4:**
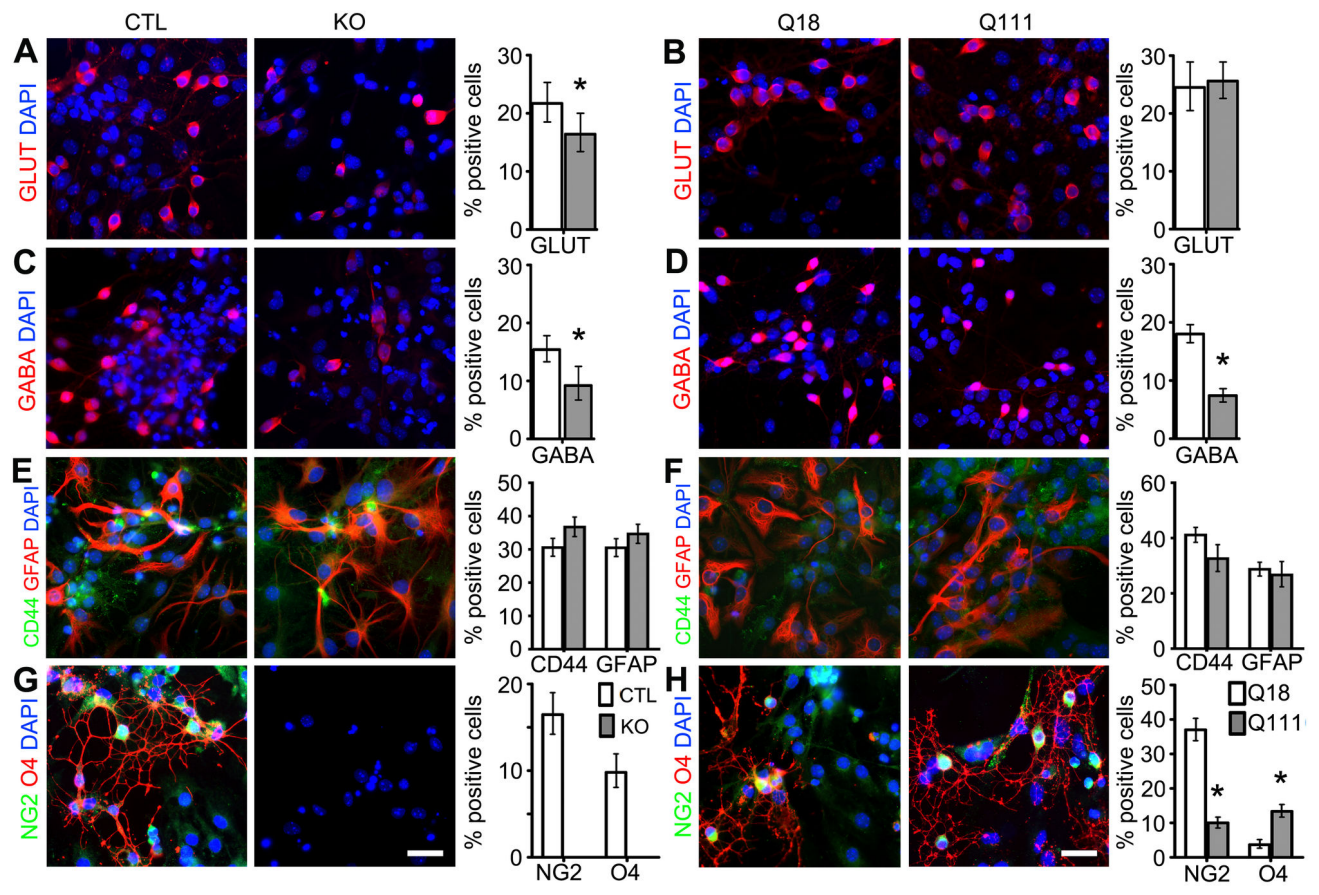
Htt is required for selective neuronal differentiation and oligodendrogliogenesis, whereas mHtt differentially impairs these processes. (A, B) Immunofluorescence analysis and quantification of GLUT+ glutamatergic neurons in CTL versus KO and in Q18 versus Q111 ESCs (n=553, 486, 400 and 738 for CTL, KO, Q18 and Q111, respectively). (C–D) Immunofluorescence analysis and quantification of GABA+ GABAergic neurons in CTL versus KO and in Q18 versus Q111 ESCs (n=1000, 381, 2341 and 2086 for CTL, KO, Q18 and Q111, respectively). (E–F) Immunofluorescence analysis and quantification of CD44+ astrocyte progenitors and GFAP+ astrocytes in CTL versus KO and in Q18 versus Q111 ESCs (n=1125, 1036, 1257 and 920 for CTL, KO, Q18 and Q111, respectively). (G–H) Immunofluorescence analysis and quantification of NG2+ oligodendrocyte precursors and O4+ oligodendrocyte progenitor cells in CTL versus KO and in Q18 versus Q111 ESCs (n=935, 421, 869 and 1291 for CTL, KO, Q18 and Q111, respectiively). All error bars represent ±95% CI; *p<0.0001 unless otherwise noted. Scale bar = 20 µm.

Deficits during the elaboration of the three cardinal germ layers may be detrimental for the specification and maturation of tissue-specific lineages. To this end, we first investigated whether Htt plays functional roles in the specification and maturation of neuronal and glial cell types, and if so, whether mHtt impairs these functions by employing established ESC differentiation experimental protocols to generate ESC-derived glutamatergic and GABAergic neurons, astrocytes and oligodendrocytes (OLs) (see Materials and Methods). As compared to the CTL cells, the number of KO ESC-derived glutamatergic and GABAergic neurons were significantly reduced (GLUT: 21.7% vs 16.5%, p-value = 0.0335; GABA: 15.4% vs 9.2%, p-value < 0.0001; Figure 4A and C). By contrast, only the number of GABAergic but not glutamatergic neurons was significantly reduced in Q111 versus Q18 cells (GLUT: 25.6% vs 24.5%; GABA: 18.0% vs 7.4%, p-value < 0.0001; Figure 4B and D). In addition, there was no difference in the number of ESC-derived GFAP+ astrocytes generated in either KO versus CTL or Q111 versus Q18 cell lines (Figure 4E-F). Interestingly, there was no elaboration of NG2+ OL precursors and O4+ OL progenitors in KO cells as compared to CTL cells (Figure 4G). By contrast, there was an increase in the number of O4+ OL progenitors in concert with a reduction in the number of NG2+ OL precursors in Q111 as compared to Q18 cells (NG2: 10.0% vs 37.1%, p-value < 0.0001; O4: 13.4% vs 3.7%, p-value < 0.0001; Figure 4H). Overall, these observations suggest that Htt plays important functions in glutamatergic and GABAergic neurogenesis as well as oligodendrogenesis, whereas mHtt selectively impairs GABAergic neuronal specification and oligodendrocyte maturation.

### Htt is required for the differentiation of endodermal- and mesodermal-derived organ-specific cell types, whereas mHtt promotes precocious differentiation of these cell types

**Figure 5:**
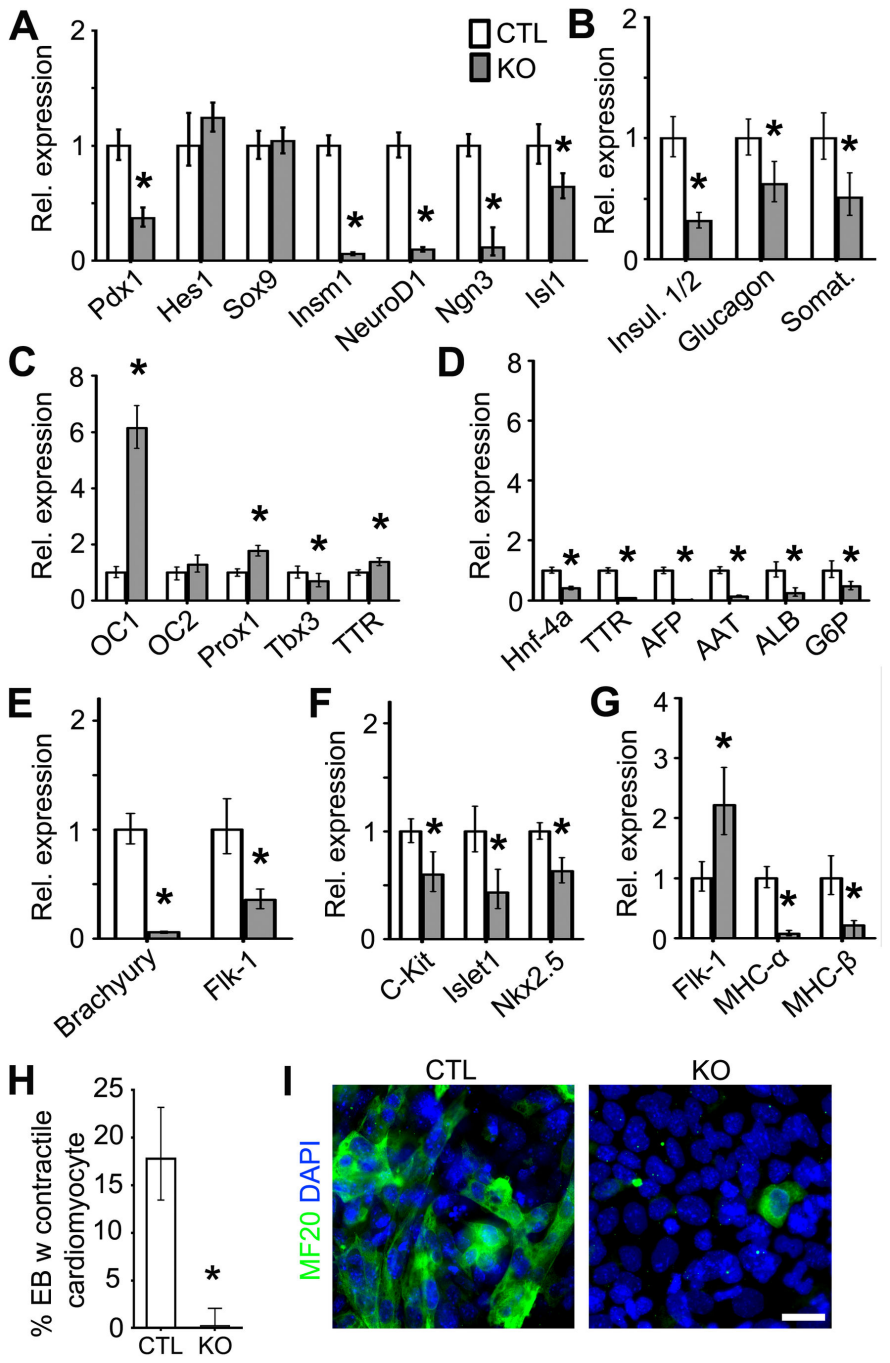
Htt is required for the specification and maturation of organ-specific lineage species. (A–B) QPCR expression analysis of markers representing pancreatic progenitors and mature pancreatic species, respectively, during pancreatic differentiation of CTL and KO ESCs. (C–D) QPCR expression analysis of markers representing early hepatoblasts and mature hepatocytes, respectively, during hepatic differentiation of CTL and KO ESCs. (E–G) QPCR expression analysis of markers representing early cardiomyocyte progenitors and mature contractile cardiomyocytes, respectively, during cardiomyocyte differentiation of CTL and KO ESCs. (H–I) Quantification of EBs with contractile cardiomyocytes and immunofluorescence analysis of MF20 expression of CTL and KO ESCs in response to cardiomyocyte differentiation. All error bars represent ±95% CI; *p<0.0001 unless otherwise noted. Scale bar = 20 µm.

**Figure 6:**
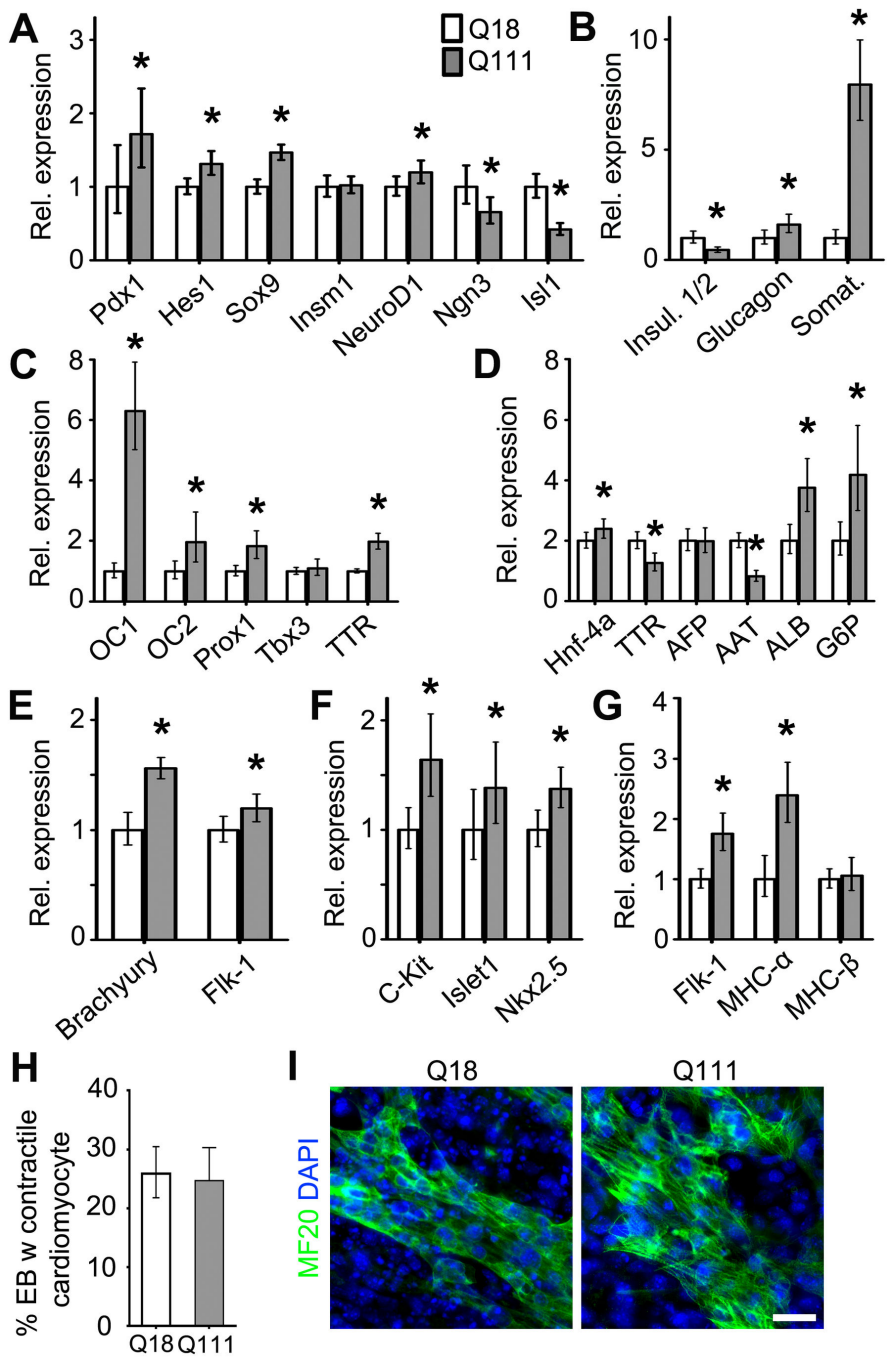
mHtt differentially alters the specification and maturation of organ-specific lineage species. (A–B) QPCR expression analysis of markers representing pancreatic progenitors and mature pancreatic species, respectively, during pancreatic differentiation of Q18 and Q111 ESCs. (C–D) QPCR expression analysis of markers representing early hepatoblasts and mature hepatocytes, respectively, during hepatic differentiation of Q18 and Q111 ESCs. (E–G) QPCR expression analysis of markers representing early cardiomyocyte progenitors and mature contractile cardiomyocytes, respectively, during cardiomyocyte differentiation of Q18 and Q111 ESCs. (H–I) Quantification of EBs containing contractile cardiomyocytes and immunofluorescence analysis of MF20 in CTL and KO ESCs in response to cardiomyocyte differentiation. All error bars represent ±95% CI; *p<0.0001 unless otherwise noted. Scale bar = 20 µm.

Given our observation that loss of Htt and the presence of mHtt enhanced the specification of endodermal cell fate, we next examined whether Htt plays a role in endodermal-derived lineage differentiation. As pancreatic cellular lineages are derived from the endoderm, we used a previously described ESC pancreatic differentiation protocol to generate ESC-derived pancreatic lineages *in vitro* [23]. As compared to the CTL cells, expression analysis of KO cell types revealed significant downregulation of genes involved in the specification of pancreatic progenitors (*Pdx1*_*Fc*_: 0.369, p-value < 0.001; *Insm1*_*Fc*_: 0.058, p-value < 0.001; *NeuroD1*_*Fc*_: 0.096, p-value < 0.001; *Ngn3*_*Fc*_: 0.114, p-value < 0.001; *Islet1 *_*Fc*_: 0.642, p-value = 0.004) and in the maturation of endocrine pancreatic cell types (*Insulin*_*Fc*_: 0.316, p-value < 0.001; *Glucagon*_*Fc*_: 0.620, p-value = 0.009; *Somatostatin*_*Fc*_: 0.509, p-value = 0.001) (Figure 5A and B). By contrast, expression analysis of the corresponding Q111 versus Q18 pancreatic lineage genes revealed upregulation of *Pdx1* (Fc: 1.719, p-value = 0.009), *Hes1* (Fc: 1.319, p-value = 0.001), *Sox9* (Fc: 1.463, p-value < 0.001), *NeuroD1* (Fc: 1.193, p-value = 0.029), *Glucagon* (Fc: 1.602, p-value = 0.014) and *Somatostatin* (Fc: 7.942, p-value < 0.001), and downregulation of *Neurog3* (Fc: 0.656, p-value = 0.028), *Isl1* (Fc: 0.416, p-value < 0.001) and *Insulin1/2* (Fc: 0.458, p-value < 0.001) (Figure 6A and B). Since hepatic lineages are also derived from a common endodermal progenitor [24], we next assessed whether Htt plays a role in the generation of ESC-derived hepatoblasts and mature hepatocytes [25]. Interestingly, compared to CTL ESCs, hepatic differentiation of KO ESCs revealed an upregulation of genes involved in the specification of hepatoblasts, *Onecut-1* (*OC1*) (Fc: 6.137, p-value < 0.001), *Prox1* (Fc: 1.770, p-value < 0.001) and *transthyretin* (*TTR*) (Fc: 1.383, p-value < 0.001) and downregulation of *Tbx3* (Fc: 0.691, p-value < 0.001) (Figure 5C). In addition, all hepatocyte maturation genes were significantly downregulated, including *Hnf-4A* (Fc: 0.399, p-value < 0.001), *TTR* (Fc: 0.083, p-value < 0.001)*,* alpha-fetoprotein (AFP) (Fc: 0.005, p-value < 0.001), *Alpha-1-antitrypsin* (*AAT*) (Fc: 0.132, p-value < 0.001), *albumin* (*ALB*) (Fc: 0.242, p-value < 0.001) and *glucose-6-phosphates* (*G6P*) (Fc: 0.473, p-value = 0.001) (Figure 5D). Similarly, gene expression analysis of the corresponding hepatic lineages in Q111 versus Q18 cell lines revealed significant upregulation of early hepatic specification genes, including *OC1* (Fc: 6.298, p-value < 0.001), *OC2* (Fc: 1.958, p-value = 0.001), *Prox1* (Fc: 1.826, p-value < 0.001), and *TTR* (Fc: 1.972, p-value < 0.001) (Figure 6C). However, expression analysis of hepatocyte maturation genes in Q111 versus Q18 cells exhibited differential impairments, with downregulation of *TTR* (Fc: 0.634, p-value = 0.001) and *AAT* (Fc: 0.412, p-value < 0.001) and upregulation of *ALB* (Fc: 1.872, p-value < 0.001) and *G6P* (Fc: 2.087, p-value = 0.001) (Figure 6D). These observations indicate that Htt is involved in the specification and maturation of pancreatic and hepatic cell types, whereas mHtt may differentially impair the integrity of these developmental functions.

As the formation of the mesodermal cell types is significantly impaired by both loss of Htt and the presence of mHtt, we next examined whether Htt and mHtt play specific roles in mesoderm-derived lineage differentiation by employing ESC differentiation protocols to generate ESC-derived early cardiomyocyte progenitors and mature contractile cardiomyocytes *in vitro* [26]. Expression analysis of CTL versus KO cells revealed significant downregulation of genes involved in the generation of cardiomyocyte progenitors, *Brachyury* (Fc: 0.060, p-value < 0.001), *Flk-1* (Fc: 0.356, p-value < 0.001), *c-kit* (Fc: 0.597, p-value < 0.001), *Islet1* (Fc: 0.430, p-value < 0.001), and *Nkx2.5* (Fc: 0.629, p-value < 0.001); and in the maturation of contractile cardiomyocyte, *Mhc-α* (Fc: 0.071, p-value = 0.001) and *Mhc-β* (Fc: 0.215, p-value < 0.001) (Figure 5E–G). We also observed a temporal delay in the expression of the early mesodermal marker, *Flk-1* (Figure 5G), potentially leading to a failure in the generation of contractile cardiomyocytes (Figure 5H and I). By contrast, gene expression analysis of Q111 versus Q18 cells revealed upregulation of most requisite cardiomyocyte developmental genes (*Brachyury*_*Fc*_: 1.559, p-value < 0.001; *Flk-1*_*Fc*_: 1.192, p-value = 0.042; *c-kit*_*Fc*_: 1.639, p-value < 0.001; *Nkx2.5*_*Fc*_: 1.375, p-value < 0.001; *Mhc-α*_*Fc*_: 2.390, p-value < 0.001; Figure 6E–G). Although Q111 and Q18 ESCs gave rise to comparable proportions of contractile cardiomyocytes, immunofluorescence analysis of Q111 ESC-derived cardiomyocytes, using an antibody against myosin heavy chain, revealed a more elongated and mature morphology as compared to those derived from Q18 ESCs (Figure 6H-I). These observations indicate that Htt is involved in the specification and maturation of cardiomyocytes, whereas mHtt further enhances these early and late developmental functions.

### Htt is involved in the regulation of components of the Notch signaling pathway during the elaboration of embryoid body-derived germ layers, whereas mHtt potentiates this signaling pathway

**Figure 7:**
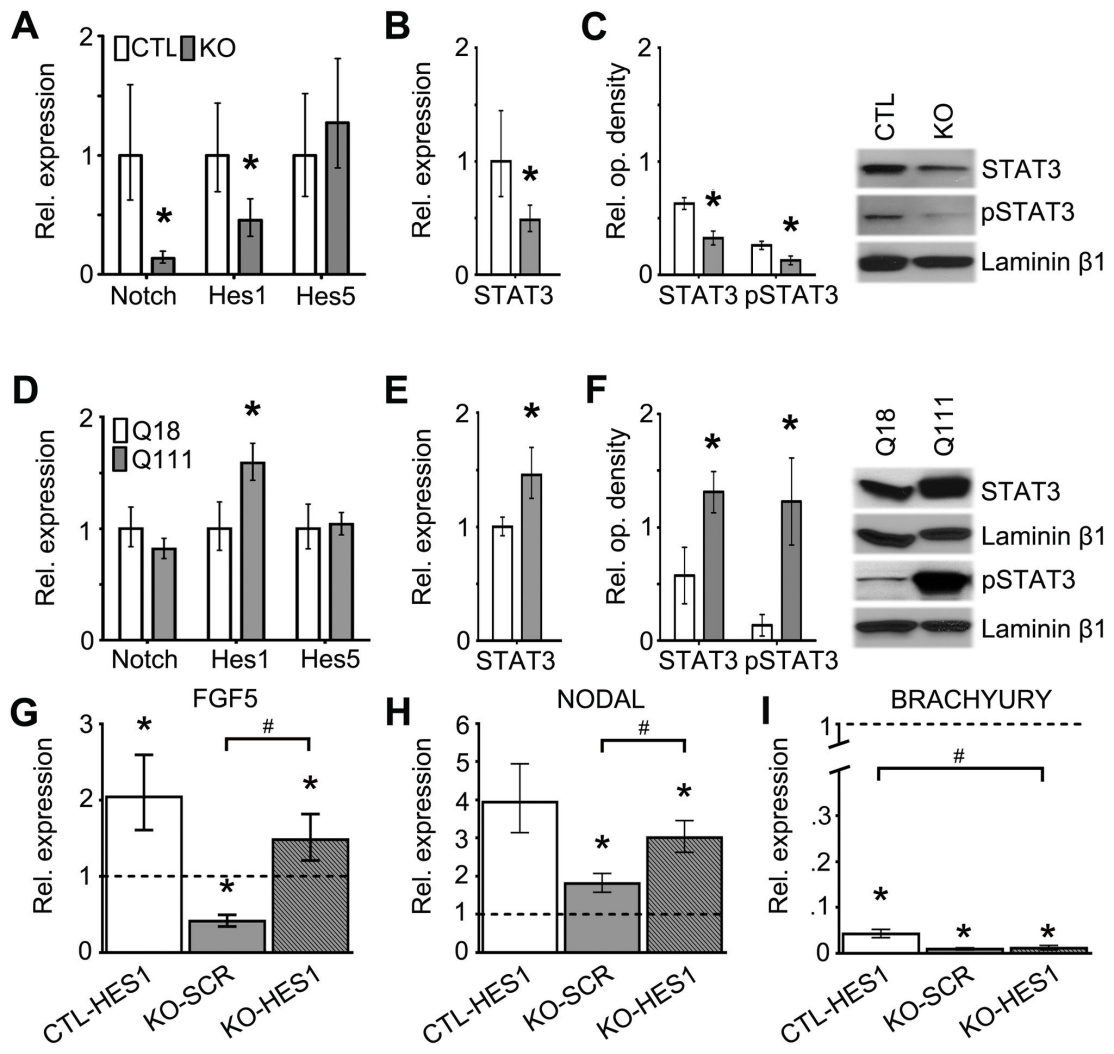
The regulation of Notch/Hes1/STAT3 signaling pathways requires Htt whereas mHtt differentially alters this process. (A) QPCR expression analysis of Notch, Hes1 and Hes5 in CTL and KO EBs at 4DIV. (B) QPCR expression analysis of STAT3 in CTL and KO EBs at 4DIV. (C) Quantification of protein levels of non-phosphorylated and phosphorylated STAT3 measured by Western blot analysis in CTL and KO EBs at 4DIV. Error bars represent ± SEM; *p<0.05. (D) QPCR expression analysis of Notch, Hes1 and Hes5 in Q18 and Q111 EBs at 4DIV. (E) QPCR expression analysis of STAT3 in Q18 and Q111 EBs at 4DIV. (F) Quantification of protein levels of non-phosphorylated and phosphorylated STAT3 measured by Western blot analysis in Q18 and Q111 EBs. Error bars represent ± SEM; *p<0.05. (G–I) QPCR expression analysis of the germ layer markers, FGF5, NODAL and BRACHYURY in CTL and KO Hes1-overexpression EBs. Dotted lines refer to expression levels of control ESCs expressing the scrambled construct, CTL-SCR. Error bars represent ±95% CI. *p<0.0001 unless otherwise noted, as compared to CTL-SCR; ^#^ p<0.0001 unless otherwise noted, as compared to KO–SCR (G, H) and CTL-HES1 (I).

Components of the Notch signaling pathway, particularly Hes1, have been implicated in the maintenance of early progenitors of many organ-specific cell types [27,28]. We therefore examined whether Htt is required for the regulation of Notch signaling cascades involved in the specification of EB-derived germ layer cells, and if so, whether mHtt alters the integrity of these developmental signaling functions. Gene expression analysis of components of the Notch signaling pathway, including *Notch*, *Hes1* and *Hes5*, in KO versus CTL EBs revealed that *Notch* and *Hes1* expression were significantly downregulated (*Notch*_Fc_: 0.139, p-value < 0.001; *Hes1*_*Fc*_: 0.454, p-value = 0.002; Figure 7A). Hes1 has also been shown to bind to and to facilitate the activation of STAT3 during the differentiation of ESC tissue-cell types [29]. Interestingly, gene expression and quantitative Western blot analysis revealed that the levels of STAT3 gene and protein expression and activation (phosphorylated STAT3 [pSTAT3]) were significantly reduced in KO versus CTL EBs (*STAT3*_*Fc*_: 0.485, p-value = 0.003; STAT3 relative optometric intensity ratio (IR): 0.33 vs 0.63, p-value = 0.01; pSTAT3_IR_: 0.13 vs 0.26, p-value = 0.041; Figure 7B-C). These findings indicate that Htt regulates the Notch signaling pathways as well as STAT3 expression and activation. Moreover, when we overexpressed *Hes1* in KO EBs (KO-HES1), as compared to CTL-SCR and KO–SCR EBs, there was significant upregulation of *FGF5* (Fc: 1.48, p-value = 0.006; Fc: 3.60, p-value < 0.001; respectively) and *Nodal* (Fc: 3.01, p-value < 0.001; Fc: 1.67, p-value < 0.001; respectively) expression. However, *Brachyury* expression in KO-HES1 EBs, as compared to CTL-SCR and CTL-HES1 EBs, still remained significantly downregulated (Fc: 0.01, p-value < 0.001; Fc: 0.25, p-value < 0.001; respectively; Figure 7G–I). These findings suggest that Htt may regulate the generation of ectodermal and endodermal cell types in response to Hes1 signaling, whereas the specification of the mesodermal cell types appeared to require Htt expression and is independent of Hes1 signaling. On the other hand, the expression of Hes1 was significantly upregulated in Q111 versus Q18 EBs (*Hes1*_*Fc*_: 1.590, p-value < 0.001), and STAT3 gene and protein expression and activation were also upregulated (*STAT3*_*Fc*_: 1.456, p-value < 0.001; STAT3_IR_: 0.58 vs 1.31, p-value = 0.0039; pSTAT3_IR_: 0.14 vs 1.22, p-value = 0.0021; Figure 7D–F). Given the differential effects of normal and mutant Htt in early embryogenesis and the complementary deficits in the Hes1/STAT3 pathways, it is likely that deregulation of Notch signaling pathways may be involved in pathogenesis of Htt-associated developmental impairments.

## Discussion

In this study, we demonstrated that Htt plays important roles in the differentiation of ESCs into ectoderm, endoderm and mesoderm, and in the subsequent specification and maturation of both neural and non-neural organ-specific lineages. In addition, we showed that Htt is involved in cell survival during germ layer specification. Our study also suggests that impairments of Notch, Hes1 and STAT3 signaling pathways may be implicated in these developmental events. Moreover, we also demonstrate that the HD pathogenic mutation differentially alters the integrity of a subset of these developmental processes without adverse effects on early embryonic cell survival.

A previous report by MacDonald et al. (2005) revealed that the loss of Htt resulted in embryonic defects ranging from head-fold involution and altered neuroectodermal gene expression to mesodermal impairments, including a shortened primitive streak and absence of the embryonic organizer [6]. However, from this important study, it was unclear whether the patterning abnormalities observed were a consequence of primary defects in either cell specification or cell survival programs. To circumvent the difficulties associated with the study of pre-implantation blastocyst *in vivo*, we decided to use ES cell culture protocols employing Htt KO and mutant Q111 ESC with appropriate control ESC lines to dissect the roles of Htt in these early developmental events. We demonstrated that the impairments in specification of mesendodermal and neuroectodermal cell types arising from the absence of Htt cannot be attenuated even in response to the strong inductive influences of the gradient morphogens, Wnt3A and RA that are essential for mediating these embryonic patterning events, indicating that Htt is involved in germ layer specification. Indeed, these observations are complementary to our previous findings of a spectrum of impairments in neural induction and early neurogenesis in knock-out Htt cell line [30]. Htt KO neural stem cells (NSCs) have also been shown to harbor impaired mobility and increase oxidative damage [31]. However, we also observed persistent and enhanced cell death in KO ESCs, which suggest that alterations in the profiles of KO EB-derived germ layer elaboration may also be secondary to differential impairments in germ layer cell survival. Our observations of enhanced cell death during the formation of ectoderm, endoderm and mesoderm from ESCs are consistent with those of two independent studies by Duyao et al. and Zeitlin et al. which reported excessive cell death in KO post-gastrulation mouse embryos [5,7]. Previous studies have also shown that Htt may regulate cell-survival by modulating the association between HIP-1 and the HIP-1 protein interactor, Hippi, which when deregulated can form pro-apoptotic Hippi-HIP-1 heterodimers that, via caspase 8, initiate the extrinsic apoptosis pathway [32,33]. Alternatively, Htt has also previously been shown to act downstream of the B-cell/Lymphoma-2 (Bcl-2)-mediated apoptosis checkpoint that regulates the activation of caspase 3 and 9 to promote apoptosis [19,20]. These Htt anti-apoptotic functions have been shown to be conserved from ancient organism such as *D. discoideum* to more evolved species including *H. sapiens* [34]. Consistently, we found that knocking down Bcl-2-associated X (BAX) in KO ESCs prevented cell death and rescued EB formation, thereby supporting previous reports that Htt may regulate cell survival through Bcl-2-mediated apoptosis signaling pathways [35,36]. However, our cell death rescue experiments did not prevent the disruption of mesodermal and ectodermal specification, confirming the fact that Htt has an important role in cell specification programs, in addition to the maintenance of cell survival.

Our study further demonstrates that Htt is involved in the elaboration of early committed non-neural, lineage-specific progenitor cell types, particularly for cardiomyocyte and pancreatic progenitors, and to a lesser extent for hepatic progenitors. Additionally, Htt has roles in the subsequent maturation of neural and non-neural lineage-committed organ-specific progenitors because the loss of Htt 1) disrupted the specification of glutamatergic and GABAergic neurons as well as the specification and maturation of oligodendrocytes and 2) severely disrupted the expression profiles of essential markers of mature hepatic, pancreatic and cardiomyocyte cell types. These cumulative developmental impairments may have resulted from the observed alterations in the integrity of Notch, Hes1 and STAT3 signaling cascades in early-stage KO-derived germ layer cells as these signaling components have been shown to be important for the maintenance and differentiation of multiple progenitors of neural, hepatic, pancreatic and cardiomyocyte lineages [37–40]. Even though Htt has not been found to directly interact with Notch, Hes1 or STAT3, our current findings do not rule out novel and indirect functional associations between these regulatory proteins. Indeed, recently reports have shown that: 1) Huntingtin interacting protein-1 (HIP1) can regulate Deltex-dependent Notch signaling for mediating neurogenesis in *Drosophila* [41] and 2) Hes1 is able to bind to STAT3 and to facilitate its expression and activation [29].

As the functions of Htt have been shown to involve many protein–protein interactions, its ablation may have severely disrupted an array of multi-protein cellular complexes, and many of these are associated with crucial regulatory networks during successive developmental stages as well as in adult life. For example, previous studies have shown that Htt is involved in modulation of the molecular complex required for degradation of β-catenin, a transcriptional regulator of the canonical Wnt signaling pathway essential for the generation of mesoderm [42]. Mouse embryos deficient for either Wnt or β-catenin completely lack the embryonic organizer and fail to generate neural structures similar to that seen in KO embryos [43,44]. These observations suggest the possibility that the multiple developmental impairments observed in KO embryos resulted from the loss of integrity of Htt-associated Wnt signaling networks. Alternatively, Htt may regulate the developmental switch between endodermal and ectodermal specification through modulating the functions of the neuron-restrictive silencing factor/RE1-silencing transcription factor (NRSF/REST), a transcriptional and epigenetic regulator of neural and non-neural fate specification that is normally sequestered in the cytoplasm by Htt [45]. The loss of Htt leads to the translocation of REST into the nucleus and thus predisposes ESC differentiation towards primitive endoderm over primitive ectoderm [46]. In addition, enhanced endodermal growth can result in precocious Nodal expression that has been shown to disrupt ESC-derived neuroectodermal differentiation in favor of the specification of endodermal and mesodermal cell types, which is consistent with our observations [47]. Alternatively, Htt has been demonstrated to be critical for homotypic interactions between neuroepithelial cells through regulation of ADAM10 activity and N-cadherin cleavage; the absence of Htt prevented proper neurulation and rosette formation [34]. Hence, the molecular processes underlying Htt developmental functions may represent novel biological mechanisms that warrant further investigations beyond the scope of this study.

Remarkably, we also demonstrated that the mutation in Htt interferes with these early developmental events. We observed an enhanced generation of neuroectodermal progenitors in the Q111 ESCs, which is complementary to our previous findings of alterations to primitive and definitive NSCs and their progeny in a Q111 cell line [30]. In addition, we observed a selective disruption in ventral forebrain GABAergic neurogenesis consistent with our previous findings of striatal developmental impairments in Q111 mice at E13.5 [11]. Finally, we found precocious elaboration of oligodendrocyte progenitors, which is consonant with previous reports of abnormalities in oligodendrocyte and white matter tracts in pre-symptomatic HD patients [48–51]. Overall, these findings of wide temporal and spatial neural developmental impairments may explain the presence of multiple foci of vulnerabilities in different brain regions reported in HD patients [52–54].

We also demonstrated that in addition to neural defects, the HD pathogenic mutation differentially impairs Htt-associated functions in non-neural cells during early embryogenesis, including alterations in the profile of representative developmental markers of liver, pancreas and cardiomyocyte cell types. Interestingly, HD is known to be associated with systemic co-morbidities affecting peripheral tissues, including those we identified in this study. For example, cardiac dysfunction associated with degenerative changes of cardiomyocytes has been reported in HD mouse models, and heart disease remains the second leading cause of death in HD patients [55–57]. Additionally, there are reports of reduced β-islet cell mass, decreased insulin secretion and altered glucose metabolism in HD mouse models and an increasing prevalence of diabetes mellitus in HD patients [58–60]. Consequently, it is possible that HD-associated impairments during early stages of embryogenesis may contribute to these non-neural pathological manifestations of HD.

Advances in employing HD-specific induced pluripotent stem cells (iPSCs) technologies have the potential to provide a useful platform to elucidate disease mechanisms, identify novel biomarkers, enhance drug screening and promote innovative therapeutic strategies [61–69]. iPSCs can be generated from various somatic cells, such as fibroblasts, via multiple reprogramming approaches, based on ESC culture technologies that require the integrity of early embryogenesis and also later stages associated with organogenesis [70]. Although a previous study reported HD-specific iPSCs did not exhibit early developmental impairments in the specification of the three cardinal germ layers [71], our findings strongly suggest that these processes are, in fact, deregulated. These differences may stem from variations in the experimental protocols utilized as a consequence of employing different types of cell lines. An alternate explanation for the discrepancies observed is the fact that the pathogenic HD mutation differentially alters components of the early embryonic developmental programs involved in iPSC generation. These considerations reinforce the need for further examination of the developmental potential and disease relevance of patient specific iPSC technologies.

Overall, our findings not only suggest that Htt is involved in the development of neural and non-neural tissues and organ systems, but also that the mutation in Htt disrupts these seminal developmental events. Hence, HD may represent the prototype of a new class of primary developmental disorders [72], with molecular and cellular impairments that may begin during early embryogenesis. The broad implications of our findings for HD pathogenesis justify additional research initiatives involving other animal models, human pathological specimens and interrogation of potential complementary pathogenic mechanisms.

## Materials and Methods

### Embryonic Stem Cell Models and Culture Paradigms

ATCC R1 wild-type embryonic stem cells (ESCs) is utilized as the control for the homozygous *htt-*knockout ESCs (KO; Hdh^ex4,5/ex4,5^). The normal *htt* knock-in Q18 ESCs, harboring a knock-in human normal exon-1 of *htt* (coding for a track of 18 glutamines) is utilized as the control for the mutant *htt* knock-in Q111 ESCs, which harbors a knock-in human mutant exon-1 of *htt* (coding for a track of 111 glutamines). The genetic background of all ESC lines is from the same mouse strain (129/Sv). These ESC lines were originally generated as a step to develop Htt KO and Q111 animal models [14–16]. The number of ESC passages was kept at 8 passages or less and karyotypic profiles were analyzed prior to experimental manipulations. ESCs were maintained in an undifferentiated state on 0.1% gelatin-coated tissue culture plates in ES cell media consisting of knockout Dulbecco’s minimal essential medium (Invitrogen, DMEM, 10313), 10% ES-qualified FBS (ATCC, SCRR-30-2020), 1X MEM nonessential amino acids (from 100x stock, Invitrogen 11140), 1X L-glutamine and antibiotics (from 100x stock, Invitrogen 10378-016), 0.1 mM 2-mercaptoethanol (Sigma, M7522), supplemented with 1000 U/ml of leukemia inhibitory factor (LIF/ESGRO; Chemicon, ESG1106). Media was changed daily and cell density was kept below 70% confluency.

### Spontaneous ESC Differentiation, Germ Layer Progenitor Cell Differentiation and Embryoid Body Formation

Spontaneous differentiation of ESCs was carried out by plating 1.0x10^4^ cells/cm^2^ on gelatin-coated tissue culture plates in ESC, Media in the absence of LIF for up to 4DIV. Differentiation of mesendodermal and neuroectodermal progenitors from ESCs were carried out as previously described [22]. Briefly, ESCs were plated at 1.5x10^4^ cells/cm^2^ on gelatin-coated tissue culture plates or poly-L-ornithine coated glass coverslips in N2B27 media for 48 hr (Pre-induction stage). After 48 hr, media is replaced with fresh N2B27 supplemented with either 500 nM retinoic acid (Sigma) or 200 ng/ml Wnt3A (R&D), and cells were cultured for a further 24-36 hr (Post-induction stage) [22]. Embryoid body differentiation was carried out by 1) plating ESCs onto non-adherent bacterial culture dishes at a density of 2-2.5 x 10^4^ cells/cm^2^, or 2) ESCs cultured as hanging drops [73] at a density of ~400 cells per drop of 20µl-30µl ESC media, for up to 10 DIV.

### Neuronal and Glial Differentiation

The neural differentiation paradigm includes selection and expansion of nestin-positive cells as previously described [74], followed by differentiation of specific neural lineages for glutamatergic neurons [75,76], GABAergic neurons [75,77], astrocytes [75], and oligodendrocytes [75]. Briefly, ESCs were cultured in flotation cultures to form EB for 4 DIV. The EBs were then plated onto adhesive tissue culture surfaces and cultured for 6-8 DIV in ITFS medium consisting of DMEM/F12 (Invitrogen, 11330) supplemented with 5µg/ml insulin (Sigma, I6634), 50µg/ml transferrin (Sigma, T1147), 30nM sodium selenium (Sigma, S5261), 5µg/ml fibronectin (Sigma, F1141), and 1x L-glutamine and antibiotics (100x stock, Invitrogen 10378-016) to select for nestin-positive cells. After selection, cells were dissociated with 0.05% trypsin/0.04% EDTA and re-plated onto either tissue culture plates or glass coverslips pre-coated with poly-DL-ornithine/laminin (Sigma P8638; BD354232) at a concentration of 1.5-2x10^5^ cells/cm^2^ in Complete Media (CM) (consisting of 500 ml DMEM/F12 supplemented with 1x N-2 (100x stock, Gibco, 17502-048) and 10mg Insulin), supplemented with 1mg/ml laminin (BD, 354232) and 10ng/ml FG2 (BD, 354060) to expand cells for 4-6 DIV. After expansion, specific neural differentiation was induced by removal of FGF2 and supplemented with the following growth factors for a further 7 DIV: 1) glutamatergic neurons; 1000 U/ml LIF, 2) astrocytes; 20 ng/ml BMP2 + 1000 U/ml LIF, 3) GABAergic neurons; 100 ng/ml SHH + 10 µg/ml BMP2, 4) dopaminergic neurons; 100 ng/ml + 100 ng/ml FGF8, 5) oligodendrocytes; 100ng/ml SHH + 10ng/ml PDGFα.

### Hepatic Differentiation

The hepatic differentiation paradigm was carried as previously described [25]. Briefly, ESCs (day 0) were maintained in flotation cultures to form EBs for 5 days in Iscove’s modified DMEM (Invitrogen, 21056-023) supplemented with 20% NCS, 1X MEM nonessential amino acids, 1X L-glutamine and antibiotics and 0.1 mM 2-mercaptoethanol. On day 5, the EBs were plated onto collagen-I (BD, 354236)-coated tissue culture plates to allow for cell growth. On day 9, the media was supplemented with 100ng/ml acidic fibroblast growth factor (aFGF, R&D, 232-FA) for another 3 days. On days 12-20, the media was supplemented with 20ng/ml hepatocyte growth factor (HGF, R&D, 2207-HG). From days 15-20, the media was also supplemented with 10ng/ml oncostatin-M (OSM, R&D, 495-MO), 10^-7^ M Dexamethasone (Sigma, D4902), 5mg/ml insulin (Sigma, I6634), 5mg/ml transferrin (Sigma, T1147), and 5µg/ml sodium selenium (Sigma, S5261). The media was changed daily. Cells were analyzed at 12 days and 20 days for early hepatic progenitors and mature hepatocytes, respectively.

### Pancreatic Differentiation

The pancreatic differentiation paradigm was carried out as previously described [23]. Briefly, ESCs were grown to form EBs for 5 days in Iscove’s modified DMEM supplemented with 20% NCS, 1X MEM nonessential amino acids, 1X L-glutamine and antibiotics and 0.1 mM 2-mercaptoethanol. After 5 DIV, EBs were plated onto gelatin-coated plates and allowed to expand for an additional 9 DIV. Thereafter, cells were dissociated using 0.05% trypsin/0.04% EDTA into single cells/small clusters and re-plated onto tissue culture plates pre-coated with Collagen I (Becton Dickinson, 4236) for an additional 19 DIV in defined medium consisting of DMEM/F12 (Invitrogen, 11330) supplemented with 20nM progesterone (Sigma, P7556), 100µM putrescine (Sigma, P5780), 1µl/ml laminin (BD, 354232), 10 mM nicotinamide (Sigma, N3376), 25 µg/ml insulin (Sigma, I6634), 30nM sodium selenium (Sigma, S5261), 50µg/ml transferrin (Sigma, T1147), B-27 media supplement (Invitrogen, 17504-044) and 1X L-glutamine and antibiotics (from 100x stock, Invitrogen 10378-016). The media was changed every two days. Cells were collected at 21 and 33 DIV for committed pancreatic progenitors and mature pancreatic species, respectively.

### Cardiomyocyte Differentiation

Cardiomyocyte differentiation was carried out as previously described [26]. Briefly, ESCs were maintained in flotation cultures to form EBs for 4 DIV in knockout Dulbecco’s minimal essential medium supplemented 10% NCS, 1X MEM nonessential amino acids, 1X L-glutamine and antibiotics and 0.1 mM 2-mercaptoethanol. Thereafter, EBs were plated onto gelatin-coated tissue culture plates and propagated for an additional 20 DIV until contractile cardiomyocytes were observed. Cells were harvested for mRNA and QPCR gene expression analysis at 4 DIV and 8 DIV, and 12 DIV for early and late cardiomyocyte progenitors, and for mature contractile cardiomyocytes, respectively.

### Immunohistochemistry, TUNEL Assay and BrdU Pulse Labeling Paradigms

Samples were fixed with 4% PFA and immunofluorescence analysis was carried out as previously described [75]. Refer to Table S1 for the antibodies employed. TUNEL analysis was performed according to the manufacturer’s protocols (Roche, 1168479591) and BrdU analysis was performed as previously described [11].

### Quantitative Real-Time PCR (QPCR) Analysis

Harvesting of RNA from samples was carried out using TRI reagent® (Molecular Research Center Inc, Cincinnati, OH, USA) according to manufacturer’s protocol. Quantification of total RNA concentration was determined using the Qubit® RNA assay kit and Qubit® 2.0 Fluorometer (Invitrogen). Single strand cDNA synthesis was performed using the High Capacity cDNA Reverse Transcription Kit® (Applied Biosystem, 4368814) following the manufacturer’s recommendations. TaqMan primers and SYBR Green probes were purchased from PE Applied Biosystems and Invitrogen service respectively (See Table S2). We utilized either TaqMan Universal PCR Master Mix® or SYBR Green Master Mix and ran samples in triplicate in the Model 7000 Real Time PCR system® (Applied Biosystems, CA, USA). We chose the housekeeping gene hypoxanthine guanine phosphoribosyl transferase 1 (*HPRT1*), based on our independent examination of the effects of mHtt on the embryonic expression of 5 widely employed house-keeping genes: GAPDH, β-actin, β2-microglobulin, Gusb and HPRT1. We found that the expression of GAPDH, β-actin and β2-microglobulin, but not Gusb and HPRT1, were significantly deregulated in the presence of mHtt. Although Gusb expression was not affected, this house-keeping gene was not suitable for our normalization as its expression level (Ct-values < 23) was far from the range of expression of target genes examined (Ct-values ranged from 27 to 29). Conversely, HPRT1 expression was not only comparable to the expression of target gene examined, but we also demonstrated that it was not affected by mHtt. All RNA samples were normalized using fluorometric procedures and levels of cDNA input were quantified and normalized using the Qubit-Invitrogen fluorometric kit for single strand DNA (previous to this quantification, heteroduplex cDNA-mRNA were treated with RNAse H). Finally, an additional normalization step was employed through quantifying the ROX fluorescence emission in each well by multiplex scanning after each PCR cycle. These additional normalization procedures significantly contributed to the achievement of a uniform, and highly replicable data results across the technical and biological replicates. Data collection and quality assessment were performed utilizing 7000 SDS 1.1 RQ Software (Applied Biosystems, CA, USA). The statistical analysis was performed using the *Pair Wise Fixed Reallocation Randomization* test provided by the [78] the Relative Expression Software Tool (REST) developed by Corbett Research [79]. Gene expression levels were reported using the relative RQ values ± 95% Confidence Interval (CI).

### Western Blot Analysis

Western blot analysis and quantification of protein levels were carried out as previously described [11]. Briefly, cells were homogenized in RIPA buffer with phosphatase inhibitor (Sigma, P0044) and protease inhibitors cocktail (Sigma, P8340) using a glass–Teflon homogenizer (10 strokes at 800 rpm) on ice, centrifuged at 900 g x 10 min and lysed in sodium dodecyl sulfate sample-loading buffer for Western blot analysis. All quantities of protein are normalized using high-precision colorimetric protein assay techniques prior to analysis.

### shBAX Viral Transduction and Hes1 Overexpression Transfection

The double short-hairpin RNA sequence specific for BAX (See Table S2) was inserted into LV-DF vector (generous gift from Dr. Joseph C. Wu [80]) using standard cloning techniques, and co-transfected with pMD2G (Addgene, cat. No. 12250) and psPAX2 (Addgene, cat. No. 12260) using the calcium phosphate precipitation technique (Clonetech, 631312) in HEK293T cells. Virus concentration was titered based on percentages of GFP^+^ ESCs and transduced with polybrene (4-8 µg/ml). GFP-positive ESCs were FACS-sorted for subsequent experimental manipulations. Conversely, *mHes1* overexpression plasmid (Addgene cat. No. 17625) was transiently transfected into ESCs using Lipofectamine^TM^ 2000 transfection reagent according to the manufacturer’s protocol (Invitrogen, 11668-027). Appropriate levels of BAX knock-down or mHes1 overexpression are reported in Figure S3.

### Statistical Analysis

All quantifications of different culture models reflect the analysis of a minimum of 10 randomly selected fields from three distinctive biological replicates. Cell counts were performed using a 40x objective (an average field area of 0.8mm^2^) and embryoid body counts were performed using either 4x or 10x objectives. Statistical comparisons were evaluated based on the type of data studied: proportions were compared with Chi-square test and the means of samples with either Mann–Whitney U test or *t*-test. Unless otherwise indicated, statistically significant differences between samples were considered using a probability of at least <0.0001 and/or <0.001.

## Supporting Information

Figure S1 **Htt is not required for the maintenance of pluripotency factor expression in undifferentiated ESCs.** (A, B) Quantification of Nanog+, Oct4+, Sox2+ (n=194 and 124 for CTL and KO, respectively) and Klf4+ (n=111 and 79 for CTL and KO, respectively), as well as KI67+ and pHisH3+ (n=127 and 173 for CTL and KO, respectively) cells in undifferentiated CTL and KO ESCs. (C) Representative images of immunofluorescence analysis of Klf4, KI67 and pHisH3 expression in undifferentiated CTL and KO ESCs. (D, E) Quantification of Nanog+, Oct4+, Sox2+ (n=93 and 131 for Q18 and Q111, respectively) and Klf4+ (n=157 and 233 for Q18 and Q111, respectively) as well as KI67+ and pHisH3+ cells (n=116 and 166 for Q18 and Q111, respectively) in undifferentiated Q18 and Q111 ESCs. (F) Representative images of immunofluorescence analysis of Klf4, KI67 and pHisH3 expression in undifferentiated Q18 and Q111 ESCs. All error bars represent ±95% CI. Scale bar = 20 µm.
(TIF)

Figure S2 **mHtt impairs the spontaneous differentiation of ESCs analogous to Htt ablation.** (A, B) Representative images of immunofluorescence analysis of the expression of the pluripotency factors, Nanog, Oct4, Sox2, Klf4 in CTL, KO, Q18 and Q111 ESCs at 4 DIV following LIF removal. (C, D) Representative images of immunofluorescence analysis of BrdU expression in CTL, KO, Q18 and Q111 ESCs at 1DIV,2DIV and 4DIV after LIF removal. (E, F) Quantification of KI67+ and pHisH3+ cells in CTL and KO ESCs at 1DIV (n=313 and 504 for CTL and KO, respectively), 2DIV (n=836 and 823 for CTL and KO, respectively) and 4DIV (n=1873 and 2020 for CTL and KO, respectively) after LIF removal. (G) Representative images of immunofluorescence analysis of the expression of the proliferation markers, KI67 and pHisH3 in CTL and KO ESCs. (H, I) Quantification of KI67+ and pHisH3+ cells in Q18 and Q111 ESCs at 1DIV (n=353 and 597 for Q18 and Q111, respectively), 2DIV (n=726 and 976 for Q18 and Q111, respectively) and 4DIV (n=1671 and 2888 for Q18 and Q111, respectively) after LIF removal. (J) Representative images of immunofluorescence analysis of the expression of the proliferation markers, KI67 and pHisH3 in Q18 and Q111 ESCs. All error bars represent ±95% CI; *p<0.0001 unless otherwise noted. Scale bar = 20 µm.
(TIF)

Figure S3 **Relative expression profiles of BAX and Hes1 in lentiviral transgenesis experiments.** (A) QPCR expression analysis of BAX in CTL-shSCR and CTL-shBAX EBs at 4DIV. (B) QPCR expression analysis of Hes1 in CTL-SCR and CTL-Hes1 EBs at 4DIV. (C) Quantification of TUNEL+ cells in CTL-shSCR, CTL-shBAX, KO-shSCR, KO-shBAX 10DIV EBs (n=3411, 3085, 2076 and 3172 for CTL-shSCR, CTL-shBAX, KO-shSCR and KO-shBAX, respectively). All error bars represent ±95% CI; *p<0.0001 unless otherwise noted.
(TIF)

Table S1 **List of antibodies utilized in the study.** All antibodies are listed with manufacturers’ names, catalogue numbers and concentration used.
(DOCX)

Table S2 **List of TaqMan probes and SYBR Green probes utilized in the study.** All TaqMan probes are listed with catalogue numbers from Applied Biosystems. All SYBR Green probes are listed with forward and reverse sequences.
(DOCX)
