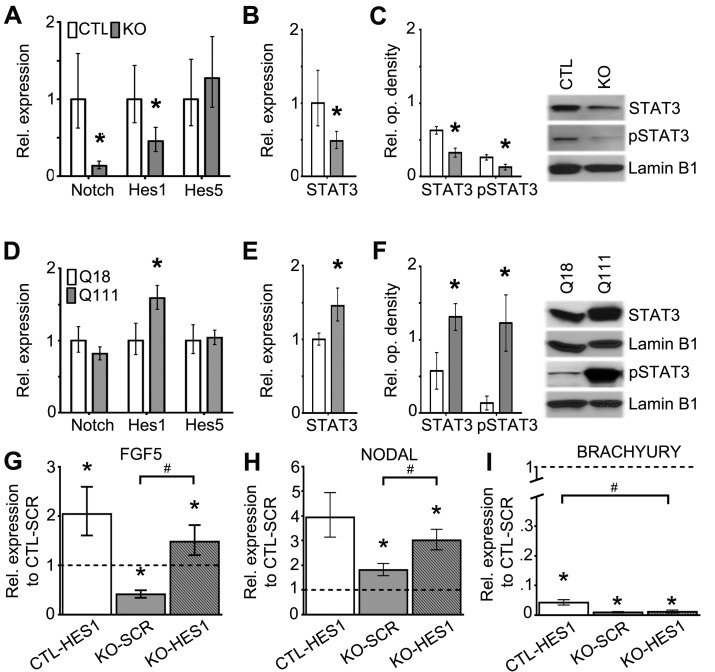# Correction: Functions of Huntingtin in Germ Layer Specification and Organogenesis

**DOI:** 10.1371/annotation/edee8dfa-6b2a-44f4-866a-098f186e27f0

**Published:** 2013-09-13

**Authors:** Giang D. Nguyen, Aldrin E. Molero, Solen Gokhan, Mark F. Mehler

There were errors in Figure 2,5, and 7. Correct versions of these Figures are available below.

Figure 2: 

**Figure:**
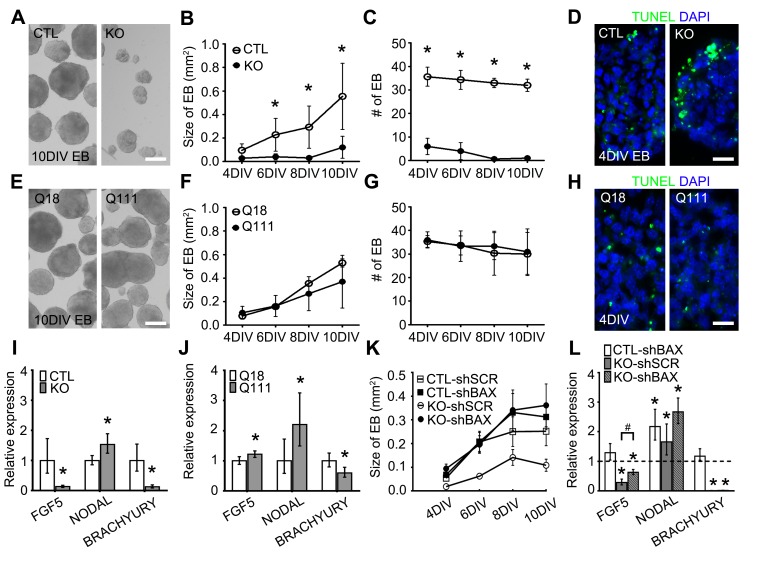


Figure 5: 

**Figure:**
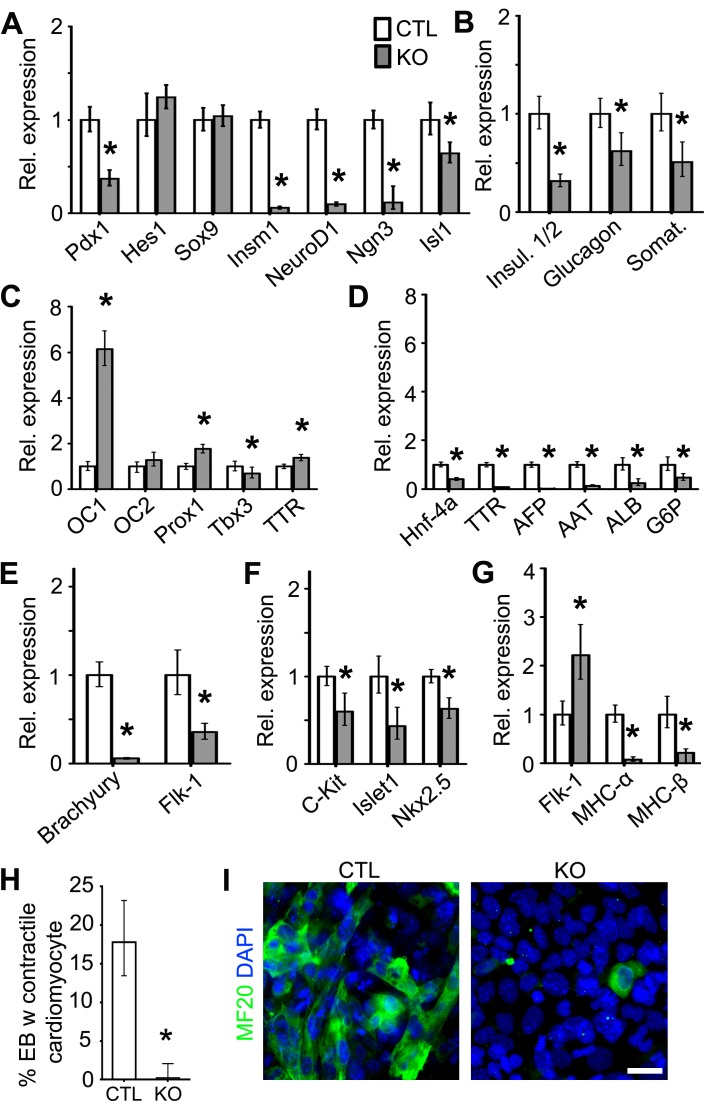


Figure 7: 

**Figure:**