# Supplementary figures and images for: Functions of Huntingtin in Germ Layer Specification and Organogenesis
Source: PLoS One. 2013 Aug 13;8(8):e72698. doi: 10.1371/journal.pone.0072698 (PMC3742581; doi:10.1371/journal.pone.0072698)

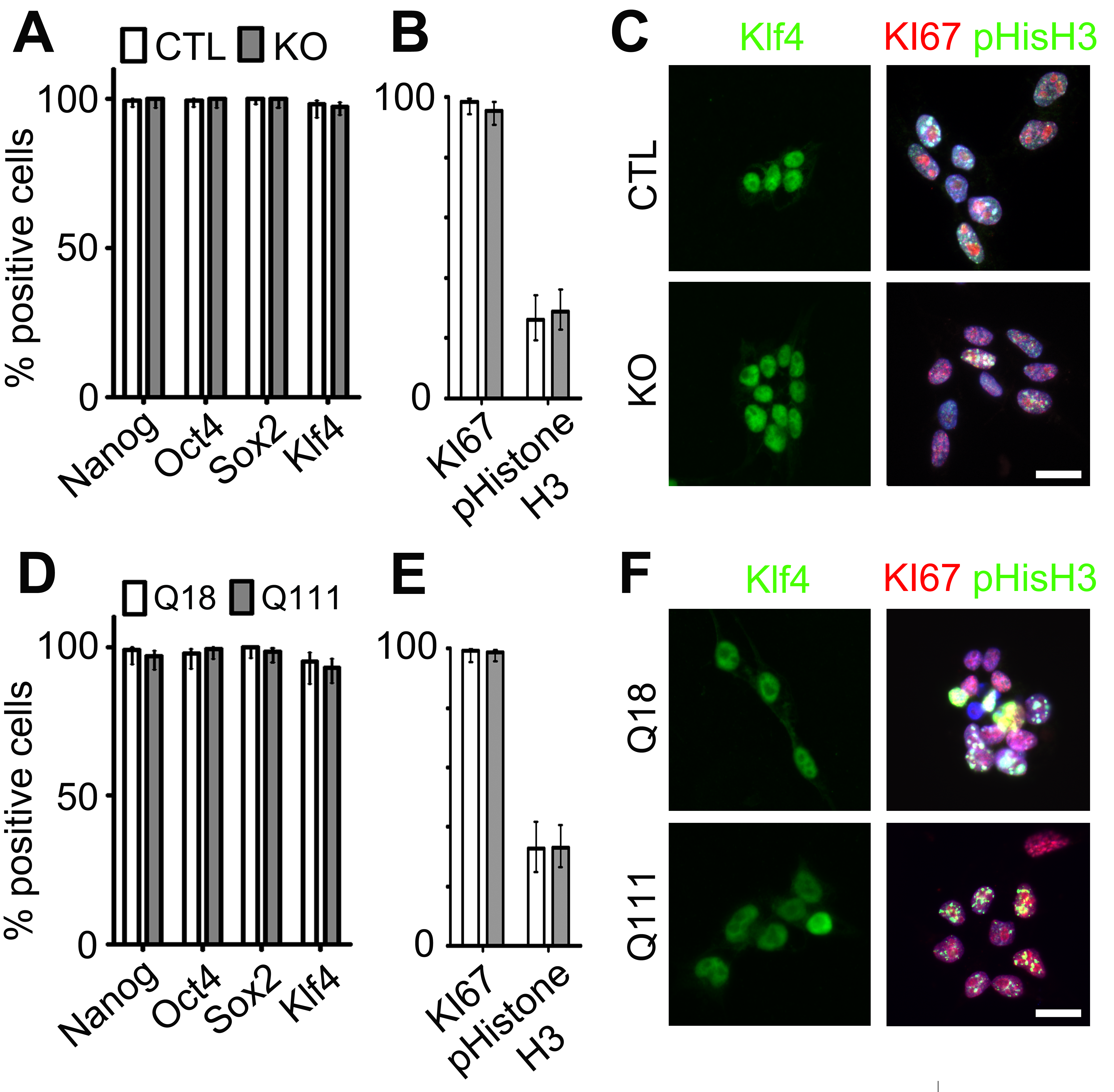

Supplement: Figure S1 — Htt is not required for the maintenance of pluripotency factor expression in undifferentiated ESCs. (A, B) Quantification of Nanog+, Oct4+, Sox2+ (n=194 and 124 for CTL and KO, respectively) and Klf4+ (n=111 and 79 for CTL and KO, respectively), as well as KI67+ and pHisH3+ (n=127 and 173 for CTL and KO, respectively) cells in undifferentiated CTL and KO ESCs. (C) Representative images of immunofluorescence analysis of Klf4, KI67 and pHisH3 expression in undifferentiated CTL and KO ESCs. (D, E) Quantification of Nanog+, Oct4+, Sox2+ (n=93 and 131 for Q18 and Q111, respectively) and Klf4+ (n=157 and 233 for Q18 and Q111, respectively) as well as KI67+ and pHisH3+ cells (n=116 and 166 for Q18 and Q111, respectively) in undifferentiated Q18 and Q111 ESCs. (F) Representative images of immunofluorescence analysis of Klf4, KI67 and pHisH3 expression in undifferentiated Q18 and Q111 ESCs. All error bars represent ±95% CI. Scale bar = 20 µm. (TIF) [file pone.0072698.s001.tif]

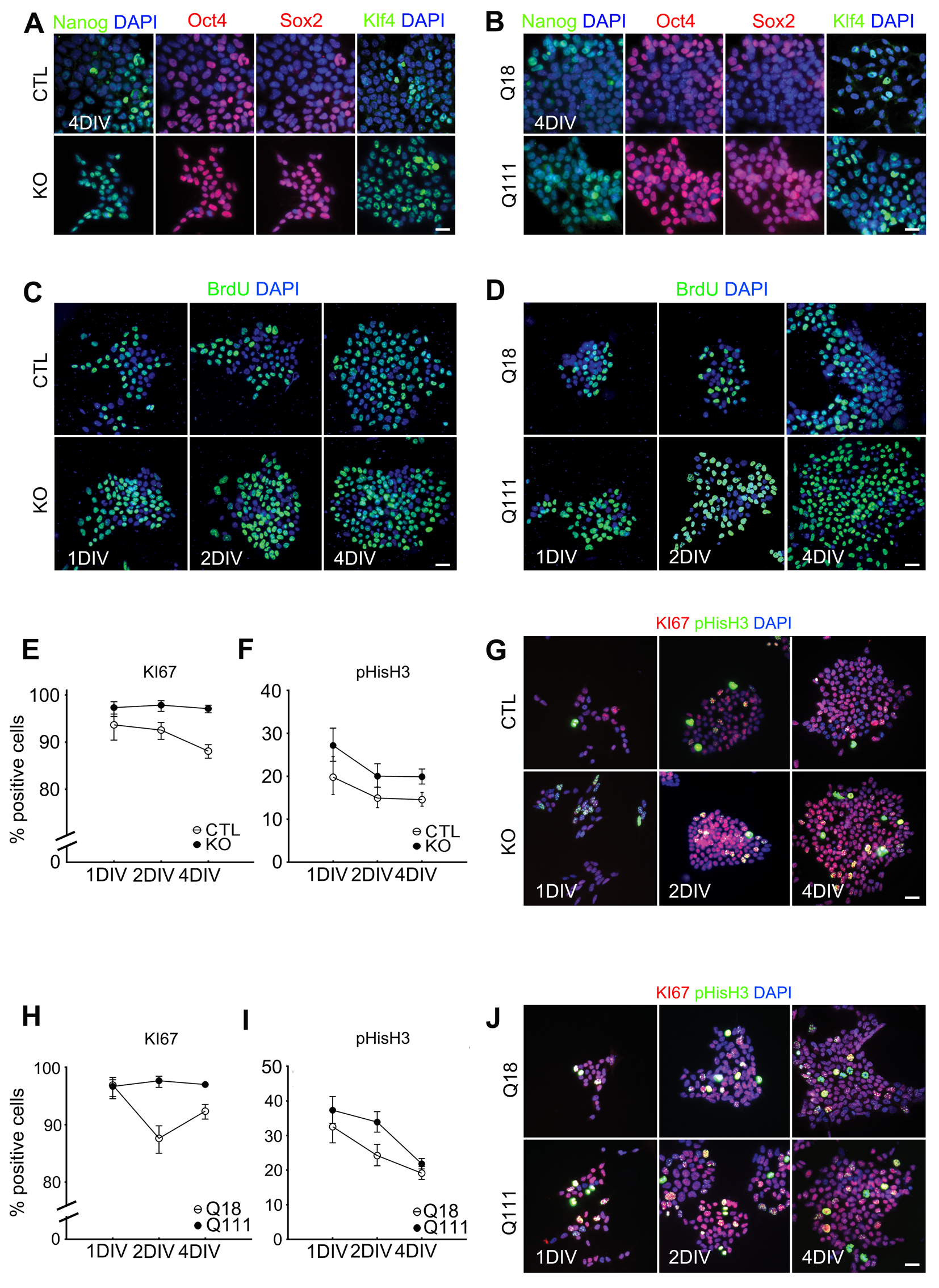

Supplement: Figure S2 — mHtt impairs the spontaneous differentiation of ESCs analogous to Htt ablation. (A, B) Representative images of immunofluorescence analysis of the expression of the pluripotency factors, Nanog, Oct4, Sox2, Klf4 in CTL, KO, Q18 and Q111 ESCs at 4 DIV following LIF removal. (C, D) Representative images of immunofluorescence analysis of BrdU expression in CTL, KO, Q18 and Q111 ESCs at 1DIV,2DIV and 4DIV after LIF removal. (E, F) Quantification of KI67+ and pHisH3+ cells in CTL and KO ESCs at 1DIV (n=313 and 504 for CTL and KO, respectively), 2DIV (n=836 and 823 for CTL and KO, respectively) and 4DIV (n=1873 and 2020 for CTL and KO, respectively) after LIF removal. (G) Representative images of immunofluorescence analysis of the expression of the proliferation markers, KI67 and pHisH3 in CTL and KO ESCs. (H, I) Quantification of KI67+ and pHisH3+ cells in Q18 and Q111 ESCs at 1DIV (n=353 and 597 for Q18 and Q111, respectively), 2DIV (n=726 and 976 for Q18 and Q111, respectively) and 4DIV (n=1671 and 2888 for Q18 and Q111, respectively) after LIF removal. (J) Representative images of immunofluorescence analysis of the expression of the proliferation markers, KI67 and pHisH3 in Q18 and Q111 ESCs. All error bars represent ±95% CI; *p<0.0001 unless otherwise noted. Scale bar = 20 µm. (TIF) [file pone.0072698.s002.tif]

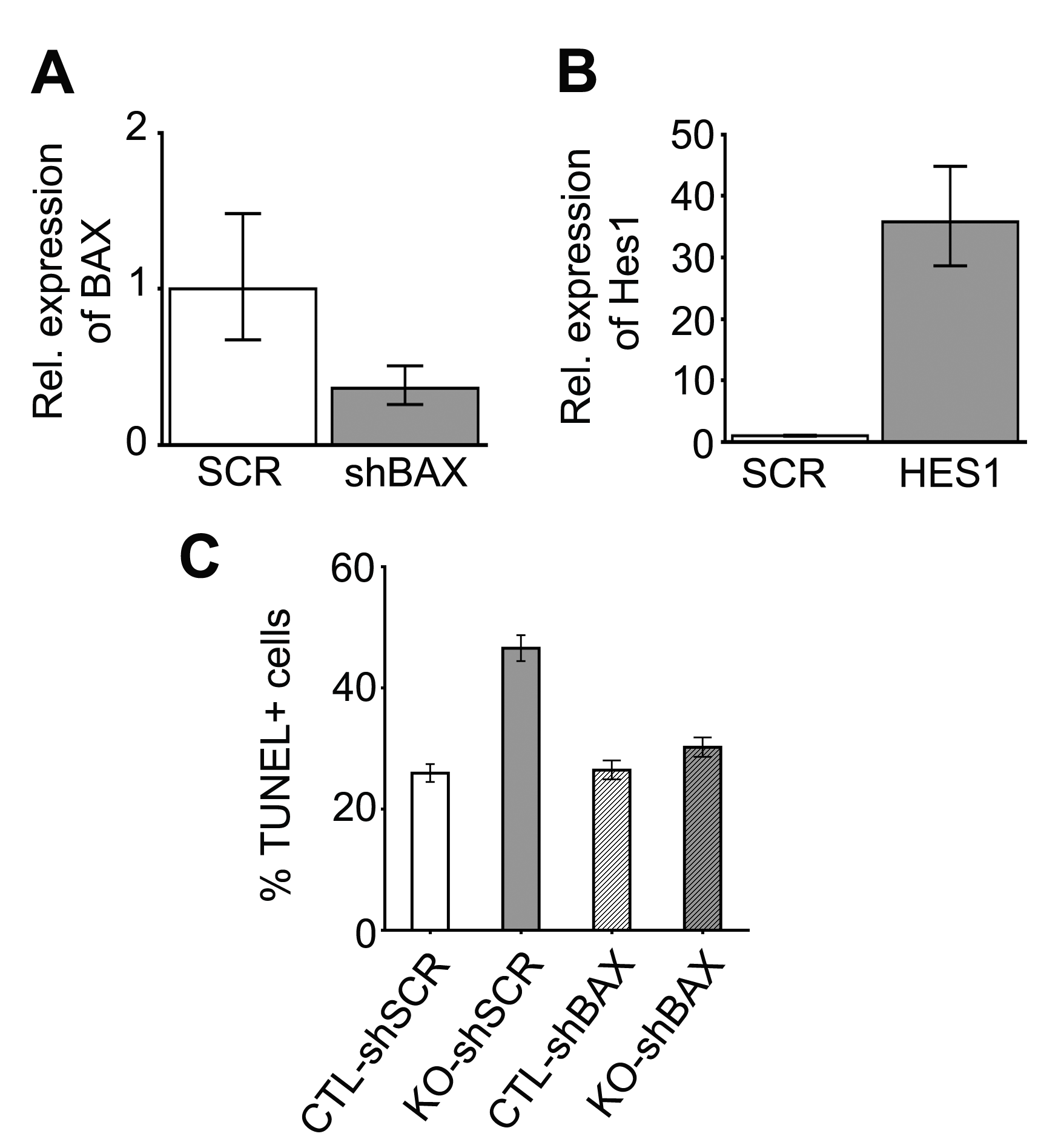

Supplement: Figure S3 — Relative expression profiles of BAX and Hes1 in lentiviral transgenesis experiments. (A) QPCR expression analysis of BAX in CTL-shSCR and CTL-shBAX EBs at 4DIV. (B) QPCR expression analysis of Hes1 in CTL-SCR and CTL-Hes1 EBs at 4DIV. (C) Quantification of TUNEL+ cells in CTL-shSCR, CTL-shBAX, KO-shSCR, KO-shBAX 10DIV EBs (n=3411, 3085, 2076 and 3172 for CTL-shSCR, CTL-shBAX, KO-shSCR and KO-shBAX, respectively). All error bars represent ±95% CI; *p<0.0001 unless otherwise noted. (TIF) [file pone.0072698.s003.tif]
