# Supplementary material for: Functions of Huntingtin in Germ Layer Specification and Organogenesis
Source: PLoS One. 2013 Aug 13;8(8):e72698. doi: 10.1371/journal.pone.0072698 (PMC3742581; doi:10.1371/journal.pone.0072698)
Supplement: Table S1 — List of antibodies utilized in the study. All antibodies are listed with manufacturers’ names, catalogue numbers and concentration used. (DOCX) [file pone.0072698.s004.docx]

**Table S1**

| Antibody | Isotype/Concentration | Manufacturer |
| --- | --- | --- |
| SSEA-1 | IgM / 1:50 | Hybridoma Bank, MC-480 |
| Nestin | IgG1 / 1:200 | B&D, 556309 |
| βIII-Tubulin | IgG2B / 1:800 | Sigma, T8660 |
| Nanog | Goat / 1:50 | R&D, AF2729 |
| Klf4 | Goat / 1:50 | R&D, AF3158 |
| Oct4 | Rabbit / 1:1000 | Abcam, AB19857 |
| Sox2 | IgG2A / 1:1000 | R&D, MAB2018 |
| GLUT | Rabbit / 1:800 | Sigma, G6642 |
| GABA | Rabbit / 1:400 | Sigma, AB2050 |
| CD44 | Rat / 1:200 | Calbiochem, 217594 |
| GFAP | IgG1 / 1:400 | Sigma, G3893 |
| NG2 | Rabbit / 1:100 | Millipore, AB5320 |
| O4 | IgM / 1:350 | Sigma, O7139 |
| MF20 | IgG1 / 1:100 | Abcam, ab15 |
| KI67 | IgG1 / 1:100 | Novo Castra, NCL-L-KI67-MM1 |
| pHisH3 | Rabbit / 1:100 | Millipore, 06-570 |
| BrdU | Rat / 1:400 | Abcam, ab6326 |
| Brachyury | Goat / 1:300 | Santa Cruz, sc-17445 |
| Sox1 | Goat / 1:300 | Santa Cruz, sc-17317 |
| STAT3 – phosphorylated | Mouse / 1:200 | Cell Signaling, 124H6 |
| STAT3 – non-phosphorylated | Rabbit / 1:200 | Cell Signaling, D3A7 |
