# Supplementary material for: Functions of Huntingtin in Germ Layer Specification and Organogenesis
Source: PLoS One. 2013 Aug 13;8(8):e72698. doi: 10.1371/journal.pone.0072698 (PMC3742581; doi:10.1371/journal.pone.0072698)
Supplement: Table S2 — List of TaqMan probes and SYBR Green probes utilized in the study. All TaqMan probes are listed with catalogue numbers from Applied Biosystems. All SYBR Green probes are listed with forward and reverse sequences. (DOCX) [file pone.0072698.s005.docx]

**Table S2**

| shBAX | | Sense: GCTCAAGGCCCTGTGCACTAAA / Anti: TTTAGTGCACAGGGCCTTGAGC |
| --- | --- | --- |
| shScrambled | | Sense: CCTAAGGTTAAGTCGCCCTCGC / Anti: GCGAGGGCGACTTAACCTTAGA |
| **TaqMan Probes** | | **Applied Biosystems catalogue number** |
| HPRT1 | | Mm00446968_m1 |
| FGF5 | | [Mm00438918_m1](https://products.appliedbiosystems.com:443/ab/en/US/adirect/ab?cmd=ABAssayDetailDisplay&assayID=Mm00438918_m1&Fs=y&adv_phrase3=EXACT&adv_phrase2=EXACT&adv_phrase1=EXACT&assayType=GE&catID=601267&batchSearchDecider=textarea&adv_kw_filter3=ALL&srchType=keyword&adv_kw_filter2=ALL&SearchRequest.Common.QueryText=14176&adv_kw_filter1=ALL&adv_query_text3=&searchType=keyword&adv_query_text2=&adv_query_text1=&uploadType=ID+List&adv_boolean3=AND&adv_boolean2=AND&adv_boolean1=AND&chkBatchQueryText=false&kwfilter=ENTREZGENE_ID&SearchRequest.Common.PageNumber=1&msgType=ABGEKeywordResults) |
| NODAL | | [Mm00443040_m1](https://products.appliedbiosystems.com:443/ab/en/US/adirect/ab?cmd=ABAssayDetailDisplay&assayID=Mm00443040_m1&Fs=y&adv_phrase3=EXACT&adv_phrase2=EXACT&adv_phrase1=EXACT&assayType=GE&catID=601267&batchSearchDecider=textarea&adv_kw_filter3=ALL&srchType=keyword&adv_kw_filter2=ALL&SearchRequest.Common.QueryText=18119&adv_kw_filter1=ALL&adv_query_text3=&searchType=keyword&adv_query_text2=&adv_query_text1=&uploadType=ID+List&adv_boolean3=AND&adv_boolean2=AND&adv_boolean1=AND&chkBatchQueryText=false&kwfilter=ENTREZGENE_ID&SearchRequest.Common.PageNumber=1&msgType=ABGEKeywordResults) |
| BRACHYURY | | [Mm01318252_m1](https://products.appliedbiosystems.com:443/ab/en/US/adirect/ab?cmd=ABAssayDetailDisplay&assayID=Mm01318252_m1&Fs=y&adv_phrase3=EXACT&adv_phrase2=EXACT&adv_phrase1=EXACT&assayType=GE&catID=601267&batchSearchDecider=textarea&adv_kw_filter3=ALL&srchType=keyword&adv_kw_filter2=ALL&SearchRequest.Common.QueryText=20997&adv_kw_filter1=ALL&adv_query_text3=&searchType=keyword&adv_query_text2=&adv_query_text1=&uploadType=ID+List&adv_boolean3=AND&adv_boolean2=AND&adv_boolean1=AND&chkBatchQueryText=false&kwfilter=ENTREZGENE_ID&SearchRequest.Common.PageNumber=1&msgType=ABGEKeywordResults) |
| Notch1 | | Mm00435245_m1 |
| Hes1 | | Mm00468601_m1 |
| Hes5 | | Mm00439311_g1 |
| **SYBR Green Probes** | | **Forward/Reverse Sequences** |
| HPRT1 | | CAGTCCCAGCGTCGTGATTA / GAATAAACACTTTTTCCAAATCCTCG |
| Pancreatic Genes | Pdx1 | AAGGCCAGTGGGCAGGAGGT / TCCTTCTCCAGCTCCAGCAGCT |
|  | Hes1 | GCAGATGACCGCCGCGCTC / CGCCCTCACACGTGGACAGG |
|  | Sox9 | GCTATCTTCAAGGCGCTGCAAGC / GCGGACCCTGAGATTGCCCAGA |
|  | Insm1 | CCGAGCGCCAAAAAGCCGAA / TCCACCGGGCCCTCCTTGAT |
|  | NeuroD1 | CAATCTTCTCTTCCGGTGCCGC / CTGGGCACTCATGACTCGCTCA |
|  | Ngn3 | AGCCTCGGACCACGAAGTGC / TCCCTCGGCAGTCACCCACT |
|  | Islet1 | GCCTCTGCAAATGGCAGCCGA / ACAGTCCGCACTCGGGTGGT |
|  | Insulin1/2 | TGGAGGACCCACAAGTGGCACA / ATGCCACGCTTCTGCTGGGC |
|  | Glucagon | ACCCCAGATCATTCCCAGCTTCC / AGTCGCTGGTGAATGTGCCCT |
|  | Somatostatin | CACCGGGAAACAGGAACTGGCC / GCTCAGCTGCCTGGGGCAAA |
| Hepatic Genes | OC1 | GCTCCGCTTAGCAGCCTGCA / CGACGTTGGACGTCTGTGAAGACC |
|  | OC2 | CAGCGCATGTCTGCCTTACGC / AGGTCCGTGAACACCAGGCG |
|  | Prox1 | CCCAGCACCGCAGAAGGACT / GCCCTTCCTGCATTGCGCTT |
|  | Tbx3 | ATCCACAGCCATGGCCTCGG / CAAGCCCTGGGAGGCCAGAA |
|  | TTR | TGTGTCTGAAGCTGGCCCCG / ACAGCCACGTCTACAGCAGGG |
|  | Hnf-4A | GCTGCAGAGCATCACCTGGCA / TGGCAGACCCTCCGAGAAGCA |
|  | TTR | CGCGGATGTGGTTTTCACAGCC / TGCTGACGACAGCCGTGGTG |
|  | AFP | AGCTGATCGACCTCACCGGGA / TGTCGGCCATTCCCTCACCACA |
|  | AAT | TCCACTGGGCATCACCCGGA / ACAGCCTTATGCACAGCCTGGC |
|  | ALB | CAACCCCACTAGCCTCTGGCA / ACTCTTGTGTGCTTCTCGGCG |
|  | G6P | CTACAGCAACAGCTCCGTGCC / TACACCTGCTGCGCCCATGG |
| Cardiomyocyte  Genes | Brachyury | ACGGCAGGAGGATGTTCCCGG / GCGGTGGTTGTCAGCCGTCA |
|  | Flk-1 | TACACGGTCATCCTCACCAA / CAAGGCTTTCTCACCGATCT |
|  | C-kit | CGTGCAGCAACAGCAATGGC / CGCGTCGCTGTCTTCTTTGCC |
|  | Nkx2.5 | CTCCTGCTTTCCCAGCCGCC / TGCAGCGCGCACAGCTCTTT |
|  | MHC-α | TCACTGCGGAAACTGAAAAC / GCCATGTCCTCGATCTTGTC |
|  | MHC-β | GTGGCAAAGTCACTGCTGAG / GCCATGTCCTCGATCTTGTC |
